# Interactions between genetics and environment shape *Camelina* seed oil composition

**DOI:** 10.1186/s12870-020-02641-8

**Published:** 2020-09-14

**Authors:** Jordan R. Brock, Trey Scott, Amy Yoonjin Lee, Sergei L. Mosyakin, Kenneth M. Olsen

**Affiliations:** 1 Department of Biology, Washington University in St. Louis, St. Louis, MO 63130 USA; 2 M.G. Kholodny Institute of Botany, National Academy of Sciences of Ukraine, 2 Tereschenkivska Street, Kyiv, 01004 Ukraine

**Keywords:** *Camelina*, Fatty acid, Environmental association, Oil content, Population structure, Phenotypic plasticity, Wild crop relatives

## Abstract

**Background:**

*Camelina sativa* (gold-of-pleasure) is a traditional European oilseed crop and emerging biofuel source with high levels of desirable fatty acids. A twentieth century germplasm bottleneck depleted genetic diversity in the crop, leading to recent interest in using wild relatives for crop improvement. However, little is known about seed oil content and genetic diversity in wild *Camelina* species.

**Results:**

We used gas chromatography, environmental niche assessment, and genotyping-by-sequencing to assess seed fatty acid composition, environmental distributions, and population structure in *C. sativa* and four congeners, with a primary focus on the crop’s wild progenitor, *C. microcarpa.* Fatty acid composition differed significantly between *Camelina* species, which occur in largely non-overlapping environments. The crop progenitor comprises three genetic subpopulations with discrete fatty acid compositions. Environment, subpopulation, and population-by-environment interactions were all important predictors for seed oil in these wild populations. A complementary growth chamber experiment using *C. sativa* confirmed that growing conditions can dramatically affect both oil quantity and fatty acid composition in *Camelina*.

**Conclusions:**

Genetics, environmental conditions, and genotype-by-environment interactions all contribute to fatty acid variation in *Camelina* species. These insights suggest careful breeding may overcome the unfavorable FA compositions in oilseed crops that are predicted with warming climates.

## Background

*Camelina sativa* (L.) Crantz is a historically important oilseed crop of Europe that has recently gained attention as a potential biofuel source [1–4] and plant factory for high-value molecules [5–9]. Much attention has been given to this species’ high seed oil content (28–43%) and its favorable fatty acid (FA) composition, which includes high levels of omega-3 FA [10, 11] and long-chain FAs that are amenable for aviation biofuels [4]. However, modern *C. sativa* varieties are characterized by low genetic diversity [12–14], which has hampered selective breeding programs in the crop. This lack of variation likely reflects a major loss of varietal diversity that occurred in the latter half of the twentieth Century, as *C. sativa* cultivation was largely abandoned throughout Europe in favor of higher-yielding oilseed rape. Knowledge of the genetic diversity and seed oil composition of *C. sativa’s* reproductively compatible wild relatives could thus be valuable for harnessing genetic resources for crop improvement.

An allohexaploid, *C. sativa*’s genome is the product of hybridization and genome duplication involving two diploid progenitor species, *C. neglecta* J. Brock et al. and *C. hispida* Boiss [15, 16]. Genomic and cytological evidence indicate that this allopolyploidization event occurred prior to *C. sativa*’s domestication from its wild progenitor, the hexaploid species *C. microcarpa* Andrz. ex DC [16, 17]. With similar genome sizes and well-documented interfertility [18, 19], crosses between *C. microcarpa* and *C. sativa* could increase genetic diversity in the crop and introduce traits for agronomic improvement. *Camelina microcarpa* has been estimated to harbor roughly twice the genetic diversity of *C. sativa* [17], which further suggests that this wild species could be valuable for breeding programs. However, little is known about *C. microcarpa* and its potential for agricultural improvement, especially regarding seed oil composition. In addition to *C. microcarpa*, other close relatives of *C. sativa* include the tetraploid species *C. rumelica* Velen., and the diploid species, *C. hispida*, *C. laxa* C. A. Mey, and *C. neglecta*. The genus comprises ~ 7–8 species in total [15, 20]. Several additional species-rank entities were recognized in the past, often based on minor morphological differences (see historical overviews in: [21, 22]; see also [17], and references therein), and some authors continue to recognize numerous narrowly-defined species (e.g., [23]).

Fatty acids are a primary seed energy source in > 80% of all flowering plant species [24]. Studies in model systems have established that both genetic and environmental factors play a role in determining their composition and total content within the seed. In maize, a genome-wide association study (GWAS) has documented that variation in kernel oil content and FA composition are controlled in part by enzymes involved in oil biosynthesis [25]. Similarly, in soybean, domestication-related genomic signatures of selection for increased oil content overlap oil content QTLs and genomic regions containing FA biosynthesis genes [26]. In Arabidopsis, a GWAS analysis identified the fatty acid desaturase gene *FAD2* as contributing to natural variation in seed FA composition [27]. Evidence for environmental influences on FA synthesis have been documented in controlled growth experiments using *Arabidopsis thaliana* (L.) Heynh. and several oilseed crop species, which have demonstrated temperature-dependent plastic responses in seed oil production [28–30]. Consistent with these findings, field trials of *C. sativa* genotypes cultivated across multiple years have revealed environmental effects in seed FA composition and oil content [31].

There is also evidence from wild species that variation in seed FA composition may play a role in local climatic adaptation. In general, higher latitudes and cooler climates are associated with decreased FA saturation in seeds; this has been documented in *Salvia*, *Helianthus* and *Arabidopsis* [27, 30, 32, 33]. Unsaturated FAs have lower melting points than saturated FAs, and while less energy-dense, are potentially more easily metabolized during germination in colder climates than saturated FAs. Climate-associated FA variation has thus been proposed to reflect an adaptive tradeoff between saturated FAs (high-energy, but less easily metabolized in colder climates) and the lower melting-point unsaturated FAs (lower-energy, but better suited to colder germination conditions) [33]. Within species, variation among populations in seed FA content may potentially reflect adaptive phenotypic plasticity across heterogeneous environments, and/or genetic factors that underlie local climatic adaptation. For the particular case of *Camelina*, the extent to which wild populations show climate-associated FA variation has not been examined, nor is it known whether such variation, if present, is attributable to genetic or environmental factors.

The present study was conducted with the goal of assessing environmental and genetic contributors to seed FA composition and content in *Camelina* species. Using wild population sampling and a combination of phenotypic and genetic assessments, we addressed the following questions: 1) Does seed FA composition differ among *Camelina* species, and to what extent is this variation associated with environmental differences in regions where they occur? 2) For the geographically widespread crop progenitor species, *C. microcarpa*, are latitude, elevation, local climate, and/or genetic substructure important predictors of seed FA composition? 3) For its domesticated derivative, to what extent can the environment alone elicit plasticity in seed FA composition? To address these questions, we analyzed the FA composition of mature seeds from wild-collected *Camelina* species, examined population structure of *C. microcarpa*, and conducted a growth chamber experiment with *C. sativa* to determine the degree of phenotypic plasticity in seed FA composition and total oil content.

## Results

### Seed oil composition differs among wild *Camelina* species

**Fig. 1:**
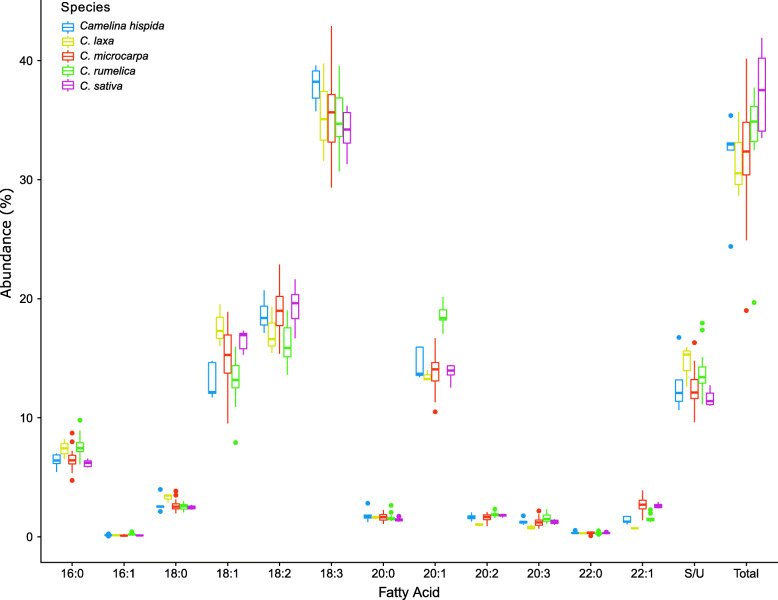
Box and whisker plot of fatty acid abundances detected in *Camelina* spp. determined via gas chromatography. Numbers of accessions measured: *Camelina hispida* (blue) *n* = 6, *C. laxa* (yellow) *n* = 3, *C. microcarpa* (red) *n* = 57, *C2019. rumelica* (green) *n* = 17, *C. sativa* (purple) *n* = 6.Values for S/U are represented as the proportion of saturated to unsaturated fatty acids. Total seed oil is represented as the percent oil relative to seed weight

Total oil content varied widely among *Camelina* seed samples (19.01–41.91%) as inferred by FAME analysis. Average seed oil content was highest in the domesticated species, *C. sativa* (37.41% ± 3.69) and lowest in *C. laxa* (31.63% ± 3.64); however, after correcting for multiple comparisons the only significant differences were between *C. sativa* and *C. microcarpa* (LMM, *p* = 0.007) and between *C. sativa* and *C. hispida* (LMM, *p* = 0.042) (Table 1). For FA composition, several FAs were found to vary widely among species (Fig. 1), such as eicosenoic acid (20:1), which was higher in *C. rumelica* relative to all other species. Erucic acid (22:1) showed the greatest relative differences among all species, with *C. microcarpa* having the highest levels at 2.66% ± 0.51 and *C. laxa* having the lowest levels at 0.74% ± 0.03 of seed oil.

**Table 1:** Linear Mixed Effects Model results for differences in total oil content between species. False discovery rate corrected *p*-values shown for each pairwise comparison; significant values at *p* < 0.05 are indicated in bold font

Species:	% Oil	***C. hispida***	***C. laxa***	***C. microcarpa***	***C. rumelica***
***C. hispida***	32.14 ± 3.95	–			
***C. laxa***	31.63 ± 3.64	0.989	–		
***C. microcarpa***	32.64 ± 3.35	0.989	0.989	–	
***C. rumelica***	34.07 ± 4.03	0.403	0.546	0.167	–
***C. sativa***	37.41 ± 3.69	**0.042**	0.108	**0.007**	0.114

We used random forest analyses to assess whether seed FA composition as a whole could be used to distinguish between species and to identify the most important FAs for differentiating them. Notably, our best random forest model was able to predict 90.8% of the species labels based on FA composition alone with a kappa of 0.825, suggesting strong predictive ability [34]. The two most informative FAs in the best model were erucic acid (22:1, mean decrease in accuracy = 119.06) and eicosenoic acid (20:1, mean decrease in accuracy = 115.45) (Supplemental Figure S1a). Although the random forest design was unbalanced due to an excess of *C. microcarpa* observations, high accuracy was nonetheless achieved for species with fewer observations; the one exception was the crop species *C. sativa*, which was not consistently distinguished from its wild progenitor, *C. microcarpa* (Supplemental Figure S2). These results indicate that while FA compositions superficially appear similar across *Camelina* species (Fig. 1), they are nonetheless readily distinguishable between species using random forest models.

### *Camelina* species occur in distinct environments

*Camelina* accessions used in this study originate from a broad geographical context, including the Caucasus (eastern Turkey, Georgia, and Armenia), Ukraine, and the eastern Rocky Mountain range of the U.S. (where *Camelina* species occur as introduced weeds). Environmental niche analyses revealed significant differences in the environments where wild *Camelina* species were found (*F* = 6.387, *p* < 0.001). Further analysis revealed that most pairwise species comparisons were also significantly different (Table 2; see also Supplemental Figure S3). At the intraspecific level, we found significant differences in environments between geographically distinct regions of *C. microcarpa* (*F* = 20.144, *p* < 0.001). This finding suggests the possibility of unique environmental niches for the geographically disparate populations of this species (see also Supplemental Figure S3). Together, these results suggest *Camelina* species largely occupy different climatic niches from each other, and that for the single species with extensive population sampling, geographical regions of that species’ range may differ environmentally as well.

**Table 2:** PERMANOVA results for environments of wild *Camelina* species. False discovery rate corrected *p*-values shown for each pairwise comparison; significant values at *p* < 0.05 are indicated in bold font

Species:	***C. hispida***	***C. laxa***	***C. microcarpa***
***C. laxa***	**0.022**	–	–
***C. microcarpa***	**0.001**	0.934	–
***C. rumelica***	0.651	**0.026**	**0.001**

### Population structure of *C. microcarpa*

**Fig. 2:**
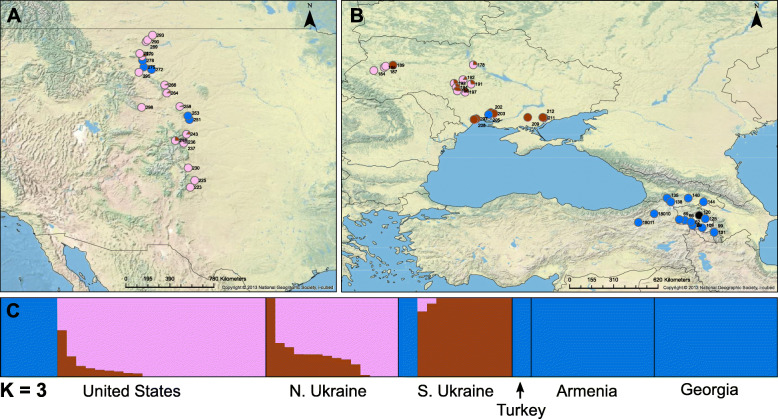
Accessions of *C. microcarpa* for which seed oil composition was analyzed are mapped as **a** collections along the Eastern Rocky Mountain range of the U.S. and **b** collections from the Caucasus and Ukraine. Colored circles represent the population structure for individuals, blue = Caucasus population, pink = northern Ukraine population, brown = southern Ukraine population, black = not genotyped/insufficient data. **c** Population structure results from ADMIXTURE analysis at K = 3 subdivided into country of origin, including 27 additional samples for which oil was not measured. Map was created under liscense using ArcGIS® software by Esri. Basemap is a source of the National Geographic Society and Esri (2019)

Cross validation scores obtained from ADMIXTURE were the lowest for K = 2 (CV error = 0.2548) and K = 3 (CV error = 0.2553), indicating that these are the two most optimal K values. At K = 2, accessions in the native range fell into two distinct subpopulations, corresponding largely to the Caucasus (eastern Turkey, Georgia, Armenia) and Ukraine; most introduced U.S. accessions fell into the Ukrainian subgroup, although several were in the Caucasus subgroup (Supplemental Figure S4). At K = 3, the Ukrainian accessions were further split into two subgroups corresponding largely to northern and southern parts of the country, with U.S. collections falling mostly in the northern Ukrainian subgroup (Supplemental Table S1). Statistical models at K = 3 provided lower AICc’s relative to K = 2; thus, K = 3 provided a stronger model fit and was chosen for subsequent analyses (Fig. 2). The Caucasus genetic subgroup showed high genetic differentiation from both the northern and southern Ukraine subgroups (F_ST_ = 0.303 and 0.314, respectively), whereas the two Ukrainian subgroups exhibited much less differentiation from each other (F_ST_ = 0.042).

Results from principal component analysis (PCA) of the genetic data were highly congruent with ADMIXTURE results (Supplemental Figure S5). Distinct clusters are evident for the Caucasus and the two Ukrainian populations, and U.S. accessions were clustered with the northern Ukrainian and Caucasus accessions. The first principal component (PC1) accounted for 65.2% of the total variation and separated the Caucasus subpopulation from the two Ukrainian groups. The second principal component (PC2) accounted for only 3.5% of the total variation and separated the northern and southern Ukrainian genotypes. These patterns of cluster separation are consistent with pairwise F_ST_ measures in the ADMIXTURE analysis.

### Population-by-environment interactions shape *C. microcarpa* oil traits

Fatty acid composition of the three *C. microcarpa* genetic subpopulations was broadly similar. Nonetheless, the northern Ukraine population showed a distinct FA profile compared to the others (Supplemental Figure S6), and random forest analysis was able to categorize these three populations based solely on FA composition with 72.6% accuracy (kappa = 0.532), providing some support for unique overall FA composition between these three groups (Supplemental Figure S1b). The distinguishable FA composition of *C. microcarpa* populations potentially suggests a genetic component to observed FA differences between populations.

We sought to determine whether population structure and environmental conditions interact to influence FA composition in *C. microcarpa*. To account for collinearity between environmental measures, a PCA was generated using all 19 BioClim variables for the local climate of each accession (Supplemental Figure S7). We used PC1 and PC2, which together accounted for 73% of the variation in environment, as variables in our models. Larger values of PC1 were associated with increased annual/diurnal range in temperature, maximum temperature of the warmest month, temperature seasonality, and isothermality, whereas lower values for PC1 were indicative of higher precipitation. On the other hand, values of PC2 were almost entirely driven by various temperature measurements such as annual mean temperature (Supplemental Figure S7).

**Fig. 3:**
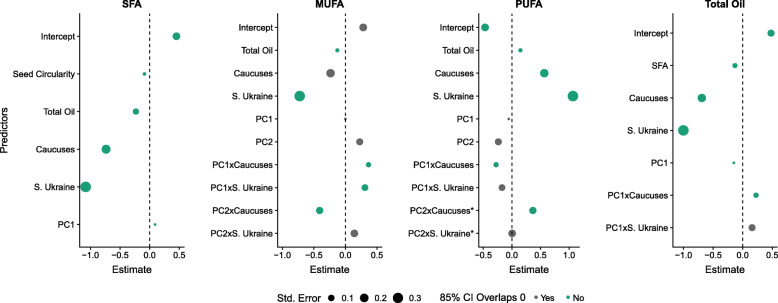
Linear Mixed Effects Model results for saturated fatty acids (SFA), mono-unsaturated fatty acids (MUFA), poly-unsaturated fatty acids (PUFA), and total oil content in *C. microcarpa*. The northern Ukraine population was used a reference population in the models, based on its difference in FA composition relative to the other genetic subpopulations (see Fig. S6). Only informative predictors are included in the figure. Colored predictors shown had confidence intervals in which the lower bound (7.5%) and upper bound (92.5%) did not overlap zero in the linear mixed effect models, and their 85% confidence intervals did not overlap zero when robust regression was performed. The 85% confidence intervals were consistent with the model selection method (see Methods)

Linear mixed modeling (LMM) uncovered interactions between population identity and these climate PCs as important predictors for FA measures. Figure 3 displays the important predictors and the size of their effects on the response variable. Moreover, robust regression showed that overall patterns were not influenced by outliers (Supplemental Table S3), and calculation of variance inflation factors showed that models did not exhibit multicollinearity among predictors. Interactions between PC1 and population identity were important predictors of mono-unsaturated fatty acids (MUFAs), poly-unsaturated fatty acids (PUFAs), and total oil, whereas interactions between PC2 and population identity were important predictors for MUFAs and PUFAs. Population identity and climate PCs individually were also important predictors for many traits independent of their interaction effects. Saturated fatty acids (SFAs) were the only group of FAs that did not include a population-by-environment interaction; however, climate (PC1) did affect SFAs in our model. Thus, larger PC1 values (associated with higher maximum monthly temperature and seasonality measures), resulted in increased SFAs; this provides some support for the hypothesis that plants in warmer climates have increased seed SFA content which may enhance germination efficiency in warm climates [33]. Across all FA measures, genetic population was found to be an important predictor six times, environment two times, and genetic population-by-environment interactions six times. These results indicate that genetics, environment, and their interactions all have an important effect on FA accumulation in *C. microcarpa* seeds (Fig. 3). In contrast, latitude and elevation were uninformative.

For total oil content, both linear mixed models and robust regression analyses indicated that SFAs and MUFAs each had a negative relationship with total oil, whereas PUFAs were positively related to the amount of total oil (Fig. 3, Supplemental Table S3). Seed circularity, used as a proxy for plant health and abiotic stress, was only informative in the SFA model, indicating that less circular seeds had higher SFAs. As with oil composition LMMs, latitude and elevation were uninformative variables that did not improve model fit for total oil content.

### Temperature elicits plasticity and GxE interactions for seed oil traits

**Fig. 4:**
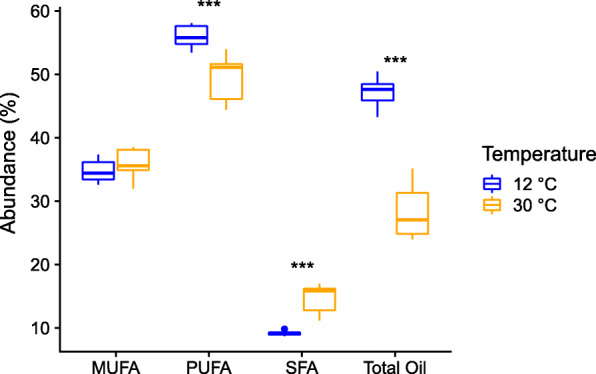
Box and whisker plot of proportions of mono-unsaturated FAs (MUFA), poly-unsaturated FAs (PUFA), saturated FAs (SFA), and total oil by seed weight in three replicates each of three *C. sativa* accessions grown at 12 °C (blue) and 30 °C (orange). *P*-values < 0.001 denoted with ***

Using the crop species, *C. sativa*, as an experimental model, we uncovered a highly plastic response for seed oil development between the cold (12 °C) and warm (30 °C) growth chamber treatments. FA composition varied greatly for each accession between treatments (Supplemental Figure S8). Mixed models showed that PUFAs and total oil decreased in the warm treatment while SFAs increased (Fig. 4, Supplemental Table S2, *p* < 0.000001), while MUFAs had a marginally significant increase in the warm temperature treatment (*p* = 0.075). The winter genotype PI 650155 displayed the lowest degree of environmental plasticity, with a 37.1% increase in total oil in cold treatment relative to warm treatment, while the spring genotypes Suneson and PI 652885 showed 77.4 and 89.9% increases, respectively, in total oil in the cold treatment (Supplemental Table S4). Taken together, these data provide strong evidence that FA composition and oil content are both environmentally plastic traits in *C. sativa*, specifically with regard to growth temperature, and that there are strong GxE effects.

## Discussion

Understanding the environmental and genetic factors that influence *Camelina* seed FA composition is a necessary first step for future plant breeding and agriculture, and can also shed light on mechanisms of local environmental adaptation in wild species. We examined the role of these factors in shaping FA composition and content. Wild *Camelina* species were found to have unique FA profiles and to largely occur in different environments (Fig. 1, Table 2, Supplemental Figures. S2 and S3). For the crop wild progenitor, *C. microcarpa*, three genetic subpopulations were discovered, which correspond to different geographical regions within the native range of the genus (Fig. 2, Supplemental Figure S5). Both local environment and subpopulation identity of *C. microcarpa* accessions were found to influence seed FA composition, including genotype-by-environment interactions (Fig. 3). Within the crop species, and when controlling for genetic background, we found that temperature alone elicits large changes in FA composition and oil content of seeds (Fig. 4). From these observations we can conclude that environment, genetics, and genotype-by-environment interactions all play a strong role in determining seed FA composition in the genus, revealing a complex path in determining seed oil characteristics. Below we discuss these findings in the context of FA variation in *Camelina* species across environments and their potential implications for oilseed agriculture.

### *Camelina* species harbor unique variation in seed oil composition

Characterizing natural variation in agriculturally relevant FAs, such as the antinutritive erucic acid (22:1), holds important relevance for crop development. While FA composition between the *Camelina* species studied herein appear superficially similar (Fig. 1), species could nearly all be readily distinguished based on FA composition using random forest models (Supplemental Figure S2). The predominant exception, the domesticated species *C. sativa* and its wild progenitor *C. microcarpa*, can likely be accounted for by the very close evolutionary relationship of these two species. The lack of differentiation in FA composition between the crop and its progenitor further suggests that FA composition was not a major target of selection during *C. sativa*’s domestication. In contrast to composition, total oil content was significantly elevated in the crop species compared to the wild progenitor (LMM, *p* = 0.007), consistent with selection for increased seed oil content during domestication. This pattern of selection on oil content but not composition during seed crop domestication has also been observed in several domesticated species relative to their predomesticates, including chickpea (*Cicer*), soybean (*Glycine*), grass pea (*Lathyrus*), common bean (*Phaseolus*), and pea (*Pisum*) [35].

Random forest analyses revealed that variation in erucic acid (22:1) was the most informative FA for distinguishing between *Camelina* species; at the intraspecific level, palmitoleic acid (16:1) was most informative for distinguishing genetically differentiated subpopulations within *C. microcarpa*, although due to its low abundance, 16:0 and 22:1 are likely more biologically informative (Supplemental Figure S1b). Fatty acids such as these, which differ significantly between evolutionarily diverged groups within *Camelina*, warrant further study. Knowledge of the genetic basis of this variation could provide an important avenue for producing a more desirable FA profile in *C. sativa* and potentially other oilseed crop species.

### Geographical and climatic distributions of *Camelina* species

While there is considerable overlap among the environments where the sampled *Camelina* species occur (Supplemental Figure S5), our data provide evidence that there is detectable environmental differentiation among some members of the genus (Table 2). For example, *C. hispida* and *C. rumelica* are present in similar environments but occur in significantly different environments from all other species (Table 2). In principle these patterns could be indicative of adaptive differences for the climates in which these species occur [33]; future population level experiments would be required to test this hypothesis.

Although the sampling for our study provided a broad representation of *Camelina* species diversity, it did not include one extant species, *C. neglecta*, as wild population samples were not available. This newly described species is known from a few collections in France [15]. Previous research has reported a unique seed FA composition and exceptionally high erucic acid content in *C. neglecta* when grown in controlled environments [36]. Additional sampling and characterization of *C. neglecta* populations may provide a promising avenue for crop improvement, as recent studies have uncovered up to two of the three subgenomes of *C. sativa* to be derived from *C. neglecta* or a close relative [16, 37]. Resynthesis of this hexaploid crop may prove possible as is the case in Brassica and the ‘Triangle of U’ [38], thus facilitating additional natural diversity and agronomic traits for crop improvement [15].

### *Camelina microcarpa* population differentiation and taxonomic identity

Population structure analyses based on genome-wide SNPs revealed three distinct genetic subpopulations of *C. microcarpa* (Fig. 2c), with a predominately Caucasus population that shows high differentiation from both northern and southern Ukrainian populations (F_ST_ > 0.30 for both pairwise comparisons). The lack of admixture between the Caucasus population and the two Ukraine populations (Fig. 2a,b), despite genotypes sometimes occurring in close proximity (e.g., in introduced U.S. locations), may indicate that these populations are divergent enough to have evolved reproductive isolating barriers that prevent admixture. Therefore, crossing experiments would be valuable to determine whether they are genetically compatible.

The genetic substructure we detect in *C. microcarpa* may also have implications for the current taxonomic ambiguities related to *C. sativa* and its congeners. *Camelina microcarpa* was formally described by Augustin Pyramus de Candolle [39] based on a specimen collected by Antoni Andrzejowski in the western and/or western-central part of Ukraine (Podillya / Podolia region). The species was provisionally named by Andrzejowski as *C. microcarpa* Andrz., but the name was not properly published before its validation by de Candolle in 1821. Thus, in our opinion, the type of the name *C. microcarpa* has not been properly designated yet, as the application of plant names at the rank of family and below requires nomenclatural types (Principle II and Art. 7.1 of the *International Code of Nomenclature for algae, fungi and plants* (*ICN*): [40]). If the lack of admixture we observe between the Ukrainian and Caucasus subpopulations is reflecting reproductive isolating barriers, a separate species designation may be warranted for one of the two groups. Given the close relationship of the Caucasus subpopulation to the crop species, this could have important implications for the taxonomic identity of the crop’s wild progenitor species. The correct type designation will be discussed in detail in a separate nomenclatural note (Mosyakin & Brock, in preparation).

A recent study on *Camelina* spp. that sampled extensively across Eurasia has revealed several ETS sequence ribotypes for *C. microcarpa* which are predominantly split between western ribotypes in Europe and eastern ribotypes in Asia [41]. However, that study did not incorporate samples from Turkey, Georgia, or Armenia, where our Caucasus population of *C. microcarpa* was predominantly found. Thus, it is unclear whether our study is missing an additional subpopulation found in Asia, or if our Caucasus population represents the same population as the Asian ‘eastern’ ribotype group. Another recent study on wild *Camelina* species also uncovered a *C. microcarpa* population that is genetically distinct from other *C. microcarpa* and *C. sativa* accessions; in this case, however, the geographical sampling suggests that the distinct genetic group corresponds to the Ukrainian populations identified in the present study.

Interestingly, introduced populations in the U.S. include representatives of at least two of these subpopulations (Caucasus, northern Ukraine) (Fig. 2a,c). These data provide, for the first-time, evidence of multiple introduction events of *C. microcarpa* as a weed into the U.S. In Canada, a recent survey of wild *Camelina* species has uncovered some individuals that are morphologically similar to *C. microcarpa* but which were discovered to be tetraploid according to flow cytometry and chromosome counts [42]. Using flow cytometry, we tested a random sample of 12 of our *C. microcarpa* collections from the eastern Rocky Mountains to determine whether any were likely to be tetraploid. All genome size measurements were consistent with hexaploidy (Supplemental Table S1). Our results thus do not provide evidence for multiple ploidy states of *C. microcarpa* within the U.S., and they eliminate the possibility that ploidy variation could be responsible for the distinct genetic differentiation and FA composition reported herein.

### Genotype and environment jointly affect oil traits in wild populations

Our study provides support for the capacity of environmental variables, including temperature and precipitation, to elicit changes to the FA composition and content of seed oil crops. In our models, large values of PC1, a proxy for maximum temperature of the warmest month and annual range of temperature, significantly increased saturated FAs (SFAs) and decreased oil content (Fig. 3). These findings agree with the notion that warm temperatures result in elevated levels of SFA [33]. Consistent with this pattern, previous studies have also revealed increased unsaturated FAs at low temperatures in flax, canola, and sunflower [28, 43, 44]. A study in *C. sativa* showed that high temperature decreases total seed oil and PUFAs, and higher precipitation improved oil content and PUFAs [45]. However, these main effects of the environmental predictors in our models were also strongly affected by interactions with population identity for MUFAs, PUFAs, and total oil; this suggests that populations could be evolving adaptations in response to climate differently. After accounting for interactions between environment and population, we identified phenotypic differences in FA measures between subpopulations, consistent with our random forest models for *C. microcarpa*. The Caucasus and southern Ukraine populations display lower SFAs with higher PUFAs and total oil when compared to the north Ukraine population (Fig. 3). Common gardens should be performed to more conclusively evaluate whether local adaptation is responsible for these differences.

An interesting outcome of our study is that environmental variables affect the same trait to different degrees between genetic populations (PCxSubpopulation interactions in Fig. 3). All FA response variables yielded at least one genotype-by-environment interaction with the exception of SFAs. A previous study did not find a significant genotype-by-environment interaction for FA composition in cultivated *C. sativa* [31]; however, the low genetic diversity in cultivated *C. sativa* [12–14] may be responsible for those results. Therefore, it is unclear whether the genotype-by-environment interactions described here are unique to *C. microcarpa* or might also exist in *C. sativa*. The ability to disentangle environment from the genetic component of seed oil composition allows for the identification of populations which may be desirable for introgression-based approaches to biofuel improvement in *C. sativa* and merits further investigation. Finally, the northern Ukraine population appears to be widespread as it occurs throughout the U.S. and Ukrainian ranges and may represent a good candidate population for introgression-based approaches to crop improvement due to its unique genotype-by-environment interactions and FA composition (lower erucic acid and higher total oil, see Supplemental Figure S6).

None of our models showed an effect of latitude or elevation on seed oil traits. These findings contradict observations in other systems such as *Helianthus* and *Arabidopsis* [30, 33] in which FA composition was found to vary across latitude. As related to previous hypotheses on local adaptation of seed oil composition, we do not see direct evidence of this in *C. microcarpa*. One potential explanation is the broader climatic and geographic sampling of *Helianthus* spp. [33], which may have revealed more coarse-scale patterns with latitude which we did not find in our study. Coarse climatic measures such as latitude and elevation are only proxies for actual environmental factors; thus, we advocate the use of finer-resolution climatic data such as those available from Bioclim [46].

### *Camelina* exhibits plasticity in seed oil composition in response to temperature

Previous research shows that high temperatures have a detrimental impact on seed oil content and composition [28, 29, 43, 44]. This may be caused by a reduced period of seed maturation, preventing developing seeds from continuing lipid biosynthesis in addition to reduced desaturation efficiency at high temperatures [29, 43]. Field trials in *C. sativa* have previously reported increases in the polyunsaturated α-linolenic acid in mild climates relative to warmer ones [47, 48]. Our growth experiment of *C. sativa* cultivated in two temperature regimes (12 °C and 30 °C) yielded strong support for environmental plasticity in seed oil content and FA composition and are consistent with previous studies. In the warm condition, lines of *C. sativa* exhibited a significant reduction in PUFAs and total seed oil and significantly elevated levels of SFAs relative to the cold (Fig. 4). Furthermore, plants had reduced levels of the omega-3, α-linolenic acid (18:3), and increased erucic acid (22:1) in the warm condition (Supplemental Figure S8). Phenotypic plasticity observed in *C. sativa* seed FA composition and oil content reported herein is also largely congruent with that observed in growth trials of *Arabidopsis thaliana* conducted at 10 °C and 30 °C [29], with the exception of 16:0, 18:2, and 22:1, which showed opposite responses to high temperatures in *C. sativa* relative to *A. thaliana*. Finally, the elevated levels of 18:1 in *C. sativa* grown at high temperature (Supplemental Figure S8) indicates that lipid biosynthesis may have been inefficient or prematurely halted, as 18:1 is a known substrate for both FA desaturation and elongation [49]. Thus, temperature alone elicits a plastic response in FA composition and oil content in *C. sativa*. Notably, these insights suggest that rising temperatures resulting from climate change could pose a detrimental effect on cultivation of *C. sativa* and other oil-seed crops through the reduction of favorable oil composition and decreased oil yield.

## Conclusions

Our study indicates that *Camelina* species often occupy specific environmental niches and that at both the species and population levels, FA compositions are distinguishable among genetically differentiated groups. Within *C. microcarpa*, environmental factors and genetic background both play a role in FA composition and total oil content, with many genotype-by-environment interactions. When controlling for genetic background, temperature alone was shown to elicit a large phenotypic shift in FAs. Thus, the present study supports the dogma that environment and genetics together determine complex phenotypes but also that populations respond to environmental conditions differentially through genotype-by-environment interactions. Considering the wide geographical distribution of *C. microcarpa*, and evidence presented herein of at least three genetically distinct populations, as well as differences in oil composition between populations, we believe that further studies on this wild predomesticate may uncover useful variation for agricultural improvement via introgression into *C. sativa*. Traditional morphology-based taxonomy should be applied in combination with molecular and experimental approaches along with broader geographical sampling to achieve a better understanding of geographical patterns, population structure, genetic relationships, and infraspecific taxonomy of the *C. sativa* and *C. microcarpa* species complex. Furthermore, insight into the effects of environment on seed oil quality in *Camelina* may be useful for future studies examining the ecological functions of seed oils and how climate change will affect wild plant populations.

## Methods

### Sample collections

To sample widely across the environmental and geographical range of wild *Camelina* populations, mature seeds were collected by J. Brock in the field from Turkey (2012, 2013, 2014), Armenia (2013), Georgia (2013), Ukraine (2017), and the United States (2018) (Supplemental Table S1). Species determinations were used from [17] and additional determinations were carried out by J. Brock with assistance from Ihsan Al-Shehbaz (Missouri Botanical Garden), with representative vouchers deposited at MO and ARIZ. All plant material was collected in compliance with institutional and international guidelines. Samples from Turkey, Armenia, and Georgia were previously described in [17] and collections were carried out in collaboration with Haceteppe University (Turkey), the National Academy of Science of Armenia, and the Georgian Academy of Sciences. All taxa collected are not regulated weeds, not listed on The International Union for Conservation of Nature’s Red List of Threatened Species (IUCN) Red List or regional Red Lists, and are not protected under the Convention on International Trade in Endangered Species of Wild Fauna and Flora (CITES).

Collections focused on *C. sativa*’s wild progenitor (*C. microcarpa*) and three closely related wild species (*C. rumelica*, *C. hispida* and *C. laxa*). Turkey and the Caucasus are likely the center of diversity for *Camelina* species and is where every extant species can be found except for the newly described species *C. neglecta* [15]. All *C. rumelica*, *C. hispida*, and *C. laxa* accessions used in this study were found in this region. The geographically widespread weedy species, *C. microcarpa*, was recovered from a broader geographical region including eastern Turkey, Georgia, Armenia, and Ukraine, as well as in the western U.S. where it is an introduced weed. The sampled range also includes areas of historic *C. sativa* cultivation, particularly Ukraine, although only one *C. sativa* accession (JRB 153, from Turkey) was found growing outside of an agricultural context; all other *C. sativa* accessions (JRB 179, 180, 181, 188, and 190) were collected from rural Ukrainian family farms where it was being cultivated on a small scale as an oilseed crop. Aside from these Ukrainian crop collections, all other accessions used in analyses were wild or weedy. No permissions were required for collecting the samples. GPS coordinates and mature seeds were collected for each accession, and geographical locations of collecting sites were mapped using ArcMap v.10.6 (ESRI, Redlands, CA, USA) and World Topo basemaps: https://www.arcgis.com/home/. The newly described species *C. neglecta* is the only extant *Camelina* species that was not sampled for the study, as wild collections were not available.

### Fatty acid phenotyping and analysis

Relative abundance and composition of seed FAs was determined for field-collected seeds of 89 *Camelina* accessions (including 57 *C. microcarpa*, 6 *C. sativa*, 17 *C. rumelica*, 6 *C. hispida*, and 3 *C. laxa*). Determinations were performed with a Fatty Acid Methyl Ester (FAME) extraction protocol slightly modified from Augustin et al. [5] as follows: Seed samples were weighed in triplicate for each accession (3–15 mg seeds per replicate). Individual replicates were then added to glass tubes with screw tops and ground using a glass stir rod with 1.5 mL 2.5% sulfuric acid in methanol before the addition of 500 μL toluene. An internal standard (50 μg mg^− 1^ triheptadecanoin) was added to each sample before incubation at 95 °C for 50 min. Samples were cooled to room temperature before the addition of 1 mL hexane and 1 mL 1 M NaCl followed by rapid mixing. Samples were centrifuged at 1500 rpm for 5 min, and the resulting hexane layer was transferred to glass autosampler vials. FAME analysis was performed by GC-FID on a Thermoquest Trace Ultra GC system with an Agilent HP-INNOwax column (30 m × 250 μm × 0.25 μm) using helium as the carrier gas. GC conditions were as follows: 60 °C for 1 min, increasing to 185 °C at a rate of 40 °C min^− 1^, increasing to 235 °C at a rate of 5 °C min^− 1^ followed by a 5 min hold. FAME species were identified by retention time compared to known standards, and relative FA abundance was determined by individual peak area divided by total area of all peaks (Supplemental Table S1). Total FAME abundance was quantified relative to the triheptadecanoin internal standard to estimate total seed oil content. FA values for all accessions were based on all three technical replicates except for accession JRB_275, where one replicate was excluded due to instrument peak integration errors.

A random forest analysis [50] was performed with *randomForest* in R to determine whether seed FA composition could be used to predict species and identify the major FA predictors. FA composition was defined by the relative abundance of 12 FAs present in seed oils. The random forest algorithm draws subsamples of the data with subsets of the total FA profile and generates decision trees for prediction. This process is bootstrapped to generate a model with better predictive ability than individual decision trees. A subset of the FA composition data (70% of the total) was used as a training set to create 5000 trees. Accessions were divided between training and test datasets so that the model had to predict completely novel accessions. To improve model fit in the face of unbalanced designs, the random forest algorithm was implemented with stratified sampling such that only one technical replicate was included in each iteration of the algorithm. Randomly sampling five and four FA variables at each split resulted in the most accurate model for *Camelina* species and *C. microcarpa* populations, respectively. The resulting model was used to predict species in the remaining 30% of the data. We assessed models with both the accuracy of predictions on the testing set and the kappa statistic [34], using the confusionMatrix function in the caret package in R [51], which is more informative for unbalanced designs.

To assess the relationship between seed morphology, FA composition and environmental factors, we measured seeds from accessions used in FA phenotyping. Greater circularity is often an indicator of higher seed fitness in *Camelina*, where abiotically stressed plants typically exhibit lower seed circularity (J. R. Brock, unpublished observations). Seeds of each accession were imaged on a Canon LiDE110 office scanner, and images were saved at 600 dpi resolution. Image files were processed in the SmartGrain analysis software [52] for measurements of seed width, length, area, perimeter, and circularity. Because these measures are all highly corelated, we chose only circularity as a measure. Seed Detection Intensity and Nogi Detection Intensity were set to ‘rough’ to allow for maximal identification of seeds before curating each scan by hand to eliminate incorrect seed identifications. Average values for seed circularity were then used in statistical analyses.

### Environmental niche analyses

Climatic variables for population locations were generated from GPS coordinates of each individual seed collection using the WorldClim 1.4 dataset [46] at the highest available spatial resolution (~ 1 km^2^). Environmental differences between locations were then assessed using permutational multivariate analysis of variance (PERMANOVAs) [53] with Euclidean distances. We tested whether there was significant dispersion between factors, which could bias the PERMANOVA results [54], in R using the betadisper function in the vegan package [55]. Dispersion tests are similar to Levene’s test in univariate ANOVAs. For comparisons between species, we used unscaled and untransformed data because scaling and transformations resulted in significantly different dispersions. For comparisons between regions of *C. microcarpa* samples, we log transformed and scaled (by subtracting the mean and dividing by the standard deviation) the data to yield no difference in dispersion. PERMANOVAs were performed with the adonis function in the vegan package with 10,000 permutations. If a significant PERMANOVA was found, we performed pairwise comparisons with false discovery rate (FDR) corrected *p*-values. PERMANOVA results were visualized with non-metric multidimensional scaling (NMDS) plots.

### Genotyping by sequencing

The influence of population structure on intraspecific FA composition was examined for the crop wild progenitor, *C. microcarpa*, which was also the species with the most FA phenotype data. DNA was extracted from 83 accessions, including 56 of the 57 accessions phenotyped for FA composition, using either a modified CTAB DNA extraction protocol [17] or DNeasy Plant Mini Kits (Qiagen, Valencia, CA, USA). Twenty-seven additional samples without FA composition data were added from collections of J.R.B. and the USDA GRIN germplasm collection to bolster sample sizes for population structure analyses (Supplemental Table S1). Genotyping-by-sequencing (GBS) libraries were then prepared with a method modified from Elshire et al. [56] as follows: Digestion of 100 ng gDNA was performed with 0.4 μL of NEB ApeKI restriction enzyme (New England Biolabs, Ipswich, MA, USA) and incubation at 75 °C for 2 h. Ligation of adapters was conducted on digested DNA with NEB T4 ligase at 22 °C for 2 h then 65 °C for 20 min. Clean-up of the ligation was performed using 25 μL AMPure XP beads (Beckman Coulter, Brea, CA, USA), and beads were washed twice with 200 μL 75% EtOH. Final PCR on the pooled library was conducted in 8 separate reactions with 5x NEB Master Mix as follows: 95 °C for 5 min; 18 cycles of 98 °C for 30 s, 65 °C for 30 s, 72 °C for 17 s; 72 °C for 5 min. Final library clean-up was performed with 44 μL AMPure XP beads, which were washed twice with 200 μL 75% EtOH. Each library was eluted in 40 μL of 10 mM Tris-HCl pH 8.0 and quantified on a Qubit fluorometer (Life Technologies, Carlsbad, CA, USA), before pooling the four highest concentration reactions for sequencing. The final library was sequenced by Novogene (Sacramento, CA, USA) on an Illumina HiSeq 4000 sequencer (Illumina, San Diego, CA, USA) for 150 bp paired-end reads. These sequence data have been submitted to GenBank under accession numbers SRR12391781 – SRR12391863.

Raw sequence reads were processed to generate a filtered SNP dataset for population structure analyses. A modified version of the Fast-GBS program [57] was implemented to enable the use of paired-end sequencing data. Within the pipeline, Sabre (https://github.com/najoshi/sabre) was used to sort and filter barcodes, and Cutadapt [58] was used to trim reads of the barcode region. Paired-end reads were then aligned to the *C. sativa* reference genome JFZQ01 [59] using BWA. Variants were searched with Platypus variant caller (https://github.com/andyrimmer/Platypus). PLINK [60] was then used with the following conditions: Minor Allele Frequency = 0.05, Genotyping = 0.1, Missing data per individual = 0.74. This resulted in 261,529 variants from 83 samples. VCFtools was used to generate the final SNP dataset using the following parameters: Max Missing = 0.5, Minor Allele Count = 3, Minimum Quality Score = 30. One sample (JRB 120) was removed due to excessive missing genotype data; 248,195 variants were removed due to missing genotype data; 3184 variants were removed due to deviations in the Hardy-Weinberg exact test; and 1450 variants were removed based on the minor allele threshold. A total of 8700 variants in 82 accessions remained in the final strict filtering dataset.

### Population structure analysis

Genetic substructure within *C. microcarpa* was assessed with ADMIXTURE v.1.3.0 [61] on the final dataset at K values from 1 to 10 with ten iterations for each K. F_ST_ measures of genetic differentiation between subpopulations were output by ADMIXTURE. Cross validation scores were obtained from ADMIXTURE for each K value. Cross validation is used to estimate error in a predictive model, where the lowest cross validation error values from ADMIXTURE represent optimal K-values. Admixture results were displayed in pong [62]. As a complementary analysis, filtered SNPs output from PLINK were also used as input in a principal component analysis (PCA) in R to visualize clusters of genetically similar accessions.

### Genotype-by-environment interaction analyses

We investigated the effect of environment and population structure on FA composition using linear mixed effect models (LMMs) fit using maximum likelihood in the lme4 package [63]. LMMs are models used to account for variation explained by fixed effects (variables of interest) and random effects. Response variables included proportions of saturated fatty acids (SFAs), mono-unsaturated fatty acids (MUFAs), poly-unsaturated fatty acids (PUFAs), and total oil content in seeds. The two most optimal population structure outputs from the ADMIXTURE analysis (K = 2 and K = 3; see Results) were included as categorical variables. To account for correlations between the BioClim variables, we performed a principal component analysis (PCA), a way to partition variation into uncorrelated components, and included the first two components in our models as fixed effects. We included latitude and elevation in our models to investigate whether these coarse measures of environment are important when accounting for more direct environmental variation using principal components (Supplemental Table S2). Seed circularity was included as a proxy for plant health, where lower values may be indicative of abiotic stress. All continuous variables were centered by subtracting the mean and scaled by dividing by the standard deviation to control for varying scales of measurement between different variables. Accession was included as a random effect to account for variation between oil measurements of the same genotype. Total oil was included as a covariate in models of SFAs, MUFAs, and PUFAs to understand how these measures change with total oil content, while SFA was included as a covariate in the total oil model such that:

$$ Total\ oil\sim SFA+ elevation+ latitude+ seed\ circ.+ PC1\ast PC2\ast population $$

$$ SFA, MUFA, PUFA\sim total\ oil+ elevation+ latitude+ seed\ circ.+ PC1\ast PC2\ast population $$

We selected the best model using small sample size-corrected Akaike Information Criteria (AICc) and the decision tree in Leroux [64]. When confidence intervals were required to choose between models, we used 85% confidence intervals since this is consistent with model selection using AIC [65]. We ensured that models were not affected by multicollinearity by calculating variance inflation factors for all final models and assessed the influence of outliers by using robust mixed-model regression from the robustlmm package [66] presented in Supplemental Table S3.

We also used linear mixed models to test for differences between total oil content between different species. Accession was included as a random effect. *P*-values were generated with the lmerTest package using Satterthwaite’s method [67] and corrected for multiple comparisons with FDR.

### Growth chamber experiments

To assess effects of growth temperature on seed oil content and composition, three lines of *C. sativa* (Suneson, PI 650155, PI 652885) were cultivated in two controlled environment growth chambers with equivalent levels of light set at 200 μmol m^− 2^ s^− 1^. One chamber mimicked a warm climate with a constant temperature of 30 °C, while the other was a cold climate chamber with a day temperature of 12 °C and night temperature of 10 °C. Both chambers were set on 16 h day, 8 h night regimes. Accession PI 650155 is a winter variety that requires vernalization to initiate flowering. Plants from this accession were therefore exposed to an 8-week period of vernalization at 4 °C before placement in the growth chambers. Seed samples from controlled growth trials were then run in FAME analysis, with three technical replicates for each of three biological replicates per accession, per condition. FAMEs were analyzed by GC-MS on a Thermo Trace Ultra GC with Thermo ITQ-900 MS system with an Agilent HP-INNOwax column (30 m × 250 μm × 0.25 μm) using a helium carrier gas. GC method conditions were as follows: 70 °C for 7 min, increasing to 185 °C at a rate of 70 °C min^− 1^, increasing to 260 °C with a 6 min hold, decreasing to 70 °C at a rate of 120 °C min^− 1^ with a 2 min hold. FAME species and total seed oil content were determined as described above for wild seed samples (Supplemental Table S4).

Growth chamber experimental results were analyzed with mixed models using temperature treatment as a fixed factor. To account for the nested design of the study, we included a nested random term with individual plants nested within accessions. *P*-values for comparisons between SFAs, MUFAs, PUFAs, and total oil were computed using Satterthwaite’s method as described above.

## Supplementary information

**Additional file 1: Figure S1.** Random forest model importance plots for individual FAs comprising total seed oils in A) all *Camelina* species examined and B) populations of *C. microcarpa*. Mean decrease in accuracy is a measure of model accuracy decrease when individual factors are removed, with larger values indicative of more important factors.

**Additional file 2: Figure S2.** Confusion matrix from random forest model predictions of species identity based on FA composition for all *Camelina* species. Colored boxes represent the number of new observations categorized by the random forest model, such that correct determinations are represented along the diagonal, all other cells indicate incorrect determinations. Boxes are shaded such that boxes which are darker blue represent a higher proportion of the total number of samples for each species.

**Additional file 3: Figure S3.** NDMS plot of environments for different species of *Camelina*. Geographical locations of *C. microcarpa* individuals indicated with shapes as follows, circle = Caucasus, triangle = Ukraine, square = USA. Euclidean distance was used to construct the plot, stress = 0.01399579.

**Additional file 4: Figure S4.** ADMIXTURE results displayed with pong at a range of K = 1–10, with 10 iterations per K. Individuals are divided into subgroups based on country of origin.

**Additional file 5: Fig\ure S5.** PCA generated from the final SNP dataset of *C. microcarpa* accessions used in ADMIXTURE analyses. Colored dots represent individual’s country of origin, colored ellipses represent population identities as determined from ADMIXTURE.

**Additional file 6: Figure S6.** Box and whisker plot of fatty acid abundances detected in *C. microcarpa* genetic populations determined via gas chromatography.

**Additional file 7: Figure S7.** PCA generated from 19 BioClim variables determined from GPS coordinates for all accessions of *Camelina* in which seed oil composition was also analyzed. Red arrows are drawn according to weight and direction of effect for each climatic variable.

**Additional file 8: Figure S8.** Fatty acid composition of three *C. sativa* accessions in 12 °C (blue) and 30 °C (orange) growth conditions as inferred by GC-MS. Error bars ±1 SD (*n* = 9).

**Additional file 9: Table S1.** *Camelina* collections, including locality information, accession averages of relative seed fatty acid abundances, bioclimatic variables, and seed morphology metrics. Collector initials, JRB = Jordan R. Brock, AAD = Ali A. Dӧnmez, Ames = USDA GRIN germplasm collection. Population ID: Defined as the population in which each accession shows majority of similarity to according to ADMIXTURE results at K = 3. N/A = Data not available, N.D. = Not detected.

**Additional file 10: Table S2.** Results of statistical models for saturated fatty acids (SFA), mono-unsaturated fatty acids (MUFA), poly-unsaturated fatty acids (PUFA), and total oil content.

**Additional file 11: Table S3.** Robust regression results of statistical models for saturated fatty acids (SFA), mono-unsaturated fatty acids (MUFA), poly-unsaturated fatty acids (PUFA), and total oil content.

**Additional file 12: Table S4.** Gas Chromatography inferred oil phenotypes for controlled environment growth experiment of three *C. sativa* accessions. Values are averages of relative peak abundance for individual fatty acids for three technical replicates per plant. SEM = Standard error of the mean, SFA = saturated fatty acid, MUFA = mono-unsaturated fatty acid, PUFA = poly-unsaturated fatty acid, S/U = proportion of saturated to unsaturated fatty acids.

## Data Availability

The dataset supporting the conclusions of this article is available in the GenBank repository and is accessible from the Sequence Read Archive (SRA) at (https://www.ncbi.nlm.nih.gov/sra) with accession numbers SRR12391781 – SRR12391863 under BioProject ID: PRJNA655452. All other statistical and gas chromatography data are available in the supplement.

## Abbreviations

TAG: Triacylglycerol
FA: Fatty acid
FAME: Fatty acid methyl ester
FA composition: The relative proportions of all types of fatty acids (FAs) in the TAGs
SFA: Saturated fatty acid (16:0, 18:0, 20:0, 22:0)
MUFA: Mono-unsaturated fatty acid (16:1, 18:1, 20:1, 22:1)
PUFA: Poly-unsaturated fatty acid (18:2, 18:3, 20:2, 20:3)
S/U: Ratio of saturated (16:0, 18:0, 20:0, 22:0) to unsaturated fatty acids (16:1, 18:1, 18:2, 18:3, 20:1, 20:2, 20:3, 22:1)

## References

[1] Blume R, Rakhmetov D (2017). Comparative analysis of oil fatty acid composition of Ukrainian spring *Camelina sativa* breeding forms and varieties as a perspective biodiesel source. Cruciferae Newslett.

[2] Iskandarov U, Kim HJ, Cahoon EB, McCann MC, Buckeridge MS, Carpita NC (2014). *Camelina*: an emerging oilseed platform for advanced biofuels and bio-based materials. Plants and bioenergy.

[3] Moser BR (2010). Camelina (*Camelina sativa* L.) oil as a biofuels feedstock: Golden opportunity or false hope?. Lipid Technol.

[4] Shonnard DR, Williams L, Kalnes TM (2010). Camelina-derived jet fuel and diesel: sustainable advanced biofuels. Environ Prog Sustain Energy.

[5] Augustin JM, Higashi Y, Feng X, Kutchan TM (2015). Production of mono- and sesquiterpenes in *Camelina sativa* oilseed. Planta.

[6] Augustin JM, Brock JR, Augustin MM, Wellinghoff RL, Shipp M, Higashi Y, Kumssa TT, Cahoon EB, Kutchan TM (2019). Field performance of terpene-producing *Camelina sativa*. Ind Crop Prod.

[7] Bansal S, Durrett TP (2016). *Camelina sativa*: an ideal platform for the metabolic engineering and field production of industrial lipids. Biochemie.

[8] Iven T, Hornung E, Heilmann M, Feussner I (2016). Synthesis of oleyl oleate wax esters in *Arabidopsis thaliana* and *Camelina sativa* seed oil. Plant Biotechnol J.

[9] Ruiz-Lopez N, Haslam RP, Napier JA, Sayanova O (2014). Successful high-level accumulation of fish oil omega-3 long-chain polyunsaturated fatty acids in a transgenic oilseed crop. Plant J.

[10] Budin JT, Breene WM, Putnam DH (1995). Some compositional properties of Camelina (*Camelina sativa* L. Crantz) seeds and oils. J Am Oil Chem Soc.

[11] Zubr J (1997). Oil-seed crop: *Camelina sativa*. Ind Crop Prod.

[12] Luo Z, Brock J, Dyer JM, Kutchan T, Schachtman D, Augustin M, Ge Y, Fahlgren N, Abdel-Haleem H (2019). Genetic diversity and population structure of a *Camelina sativa* spring panel. Front Plant Sci.

[13] Singh R, Bollina V, Higgins EE, Clarke WE, Eynck C, Sidebottom C, Gugel R, Snowdon R, Parkin IA (2015). Single-nucleotide polymorphism identification and genotyping in Camelina sativa. Mol Breed.

[14] Vollmann J, Grausgruber H, Stift G, Dryzhyruk V, Lelley T (2005). Genetic diversity in camelina germplasm as revealed by seed quality characteristics and RAPD polymorphism. Plant Breed.

[15] Brock JR, Mandáková T, Lysak MA, Al-Shehbaz IA (2019). *Camelina neglecta* (Brassicaceae, Camelineae), a new diploid species from Europe. PhytoKeys.

[16] Mandáková T, Pouch M, Brock JR, Al-Shehbaz IA, Lysak MA (2019). Origin and evolution of diploid and allopolyploid Camelina genomes was accompanied by chromosome shattering. Plant Cell.

[17] Brock JR, Dönmez AA, Beilstein MA, Olsen KM (2018). Phylogenetics of *Camelina* Crantz. (Brassicaceae) and insights on the origin of gold-of-pleasure (*Camelina sativa*). Mol Phylogenet Evol.

[18] Martin SL, Lujan-Toro BE, Sauder CA, James T, Ohadi S, Hall LM (2018). Hybridization rate and hybrid fitness for *Camelina microcarpa* Andrz. Ex DC (♀) and *Camelina sativa* (L.) Crantz (Brassicaceae) (♂). Evol Appl.

[19] Séguin-Swartz G, Nettleton JA, Sauder C, Warwick SI, Gugel RK (2013). Hybridization between *Camelina sativa* (L.) Crantz (false flax) and north American *Camelina* species. Plant Breed.

[20] Al-Shehbaz IA, Beilstein MA (2010). Camelina. Flora of North America Editorial Committee.

[21] Sinskaja EN. 1928. The oleiferous plants and root crops of the family Cruciferae. *Bulletin of Applied Botany, Genetics and Plant Breeding* [Russian title: *Труды по прикладной ботанике, генетике и селекции*] 19: 1–648. (In Russian).

[22] Sinskaja EN, Beztuzheva AA. 1931. The forms of Camelina sativa in connection with climate, flax and man. *Bulletin of Applied Botany, Genetics and Plant Breeding* [Russian title: *Труды по прикладной ботанике, генетике и селекции*] 25: 98–200. (In Russian).

[23] Dorofeyev VI (2019). *Camelina* (Cruciferae, Brassicaceae): structure of [the] genus and [a] list of species. Vavilovia.

[24] Harwood JL. 1980. Plant acyl lipids: structure, distribution, and analysis. Lipids: structure and function. 2–55 in P. K. Stumpf, ed. Lipids: structure and function.

[25] Li H, Peng Z, Yang X, Wang W, Fu J, Wang J, Han Y, Chai Y, Guo T, Yang N (2013). Genome-wide association study dissects the genetic architecture of oil biosynthesis in maize kernels. Nat Genet.

[26] Zhou Z, Jiang Y, Wang Z, Gou Z, Lyu J, Li W, Yu Y, Shu L, Zhao Y, Ma Y (2015). Resequencing 302 wild and cultivated accessions identifies genes related to domestication and improvement in soybean. Nat Biotechnol.

[27] Branham SE, Wright SJ, Reba A, Morrison GD, Linder RC (2016). Genome-wide association study in *Arabidopsis thaliana* of natural variation in seed oil melting point: a widespread adaptive trait in plants. J Hered.

[28] Canvin DT (1965). The effect of temperature on the oil content and fatty acid composition of the oils from several oil seed crops. Can J Bot.

[29] Menard GN, Moreno JM, Bryant FM, Munoz-Azcarate O, Kelly AA, Hassani-Pak K, Kurup S, Eastmond PJ (2017). Genome wide analysis of fatty acid desaturation and its response to temperature. Plant Physiol.

[30] Sanyal A, Linder CR (2013). Plasticity and constraints on fatty acid composition in the phospholipids and triacylglycerols of *Arabidopsis* accessions grown at different temperatures. BMC Plant Biol.

[31] Vollmann J, Moritz T, Kargl C, Baumgartner S, Wagentristl H (2007). Agronomic evaluation of camelina genotypes selected for seed quality characteristics. Ind Crop Prod.

[32] Ayerza R (2010). Effects of seed color and growing locations on fatty acid content and composition of two chia (Salvia hispanica L.) genotypes. J Am Oil Chem Soc.

[33] Linder CR (2000). Adaptive evolution of seed oils in plants: accounting for the biogeographic distribution of saturated and unsaturated fatty acids in seed oils. Am Nat.

[34] Landis JR, Koch GG. The measurement of observer agreement for categorical data. Biometrics. 1977:159–74. 843571

[35] Fernández-Marín B, Milla R, Martín-Robles N, Arc E, Kranner I, Becerril JM, García-Plazaola JI (2014). Side-effects of domestication: cultivated legume seeds contain similar tocopherols and fatty acids but less carotenoids than their wild counterparts. BMC Plant Biol.

[36] Wu JR (2016). Natural trait variation for taxonomic classification and breeding potential assessment in the genus Camelina.

[37] Chadhary R, Koh CS, Kagale S, Tang L, Wu SW, Lv Z, Mason AS, Sharpe AG, Diederichsen A, Parkin IA (2020). Assessing diversity in the *Camelina* genus provides insights into the genome structure of *Camelina sativa*. *G3: Genes, Genomes*. Genetics.

[38] Nagaharu U (1935). Genome analysis in Brassica with special reference to the experimental formation of *B. napus* and Peculiar Mode of Fertilization. Jpn J Botany.

[39] de Candolle AP (1821). Regni Vegetabilis Systema Naturale, sive Ordines, genera et species plantarum secundum methodi naturalis normas digestarum et descriptarum.

[40] Turland NJ, Wiersema JH, Barrie FR, Greuter W, Hawksworth DL, Herendeen PS, Knapp S, Kusber W-H, Li D-Z, Marhold K, May TW, McNeill J, Monro AM, Prado J, Price MJ, Smith GF (2018). International Code of Nomenclature for algae, fungi, and plants (Shenzhen Code) adopted by the Nineteenth International Botanical Congress, Shenzhen, China, July 2017. Regnum Vegetabile.

[41] Čalasan AŽ, Seregin AP, Hurka H, Hofford NP, Neuffer B. The Eurasian steppe belt in time and space: phylogeny and historical biogeography of the false flax (Camelina Crantz, Camelineae, Brassicaceae). Flora. 2019;260:151477.

[42] Martin SL, Smith TW, James T, Shalabi F, Kron P, Sauder CA (2017). An update to the Canadian range, abundance, and ploidy of Camelina spp. (Brassicaceae) east of the Rocky Mountains. Botany.

[43] Harris P, James AT (1969). The effect of low temperatures on fatty acid biosynthesis in plants. Biochem J.

[44] Schulte LR, Ballard T, Samarakoon T, Yao L, Vadlani P, Staggenborg S, Rezac M (2013). Increased growing temperature reduces content of polyunsaturated fatty acids in four oilseed crops. Ind Crop Prod.

[45] Obeng E, Obour AK, Nelson NO, Moreno JA, Ciampitti IA, Wang D, Durrett TP (2019). Seed yield and oil quality as affected by Camelina cultivar and planting date. J Crop Improv.

[46] Hijmans RJ, Cameron SE, Parra JL, Jones PG, Jarvis A (2005). Very high resolution interpolated climate surfaces for global land areas. Int J Climatol.

[47] Krzyżaniak M, Stolarski MJ, Tworkowski J, Puttick D, Eynck C, Załuski D, Kwiatkowski J (2019). Yield and seed composition of 10 spring camelina genotypes cultivated in the temperate climate of Central Europe. Ind Crop Prod.

[48] Zanetti F, Eynck C, Christou M, Krzyżaniak M, Righini D, Alexopoulou E, Stolarski MJ, Van Loo EN, Puttick D (2017). Agronomic performance and seed quality attributes of Camelina (Camelina sativa L. crantz) in multi-environment trials across Europe and Canada. Ind Crop Prod.

[49] Li-Beisson Y, Shorrosh B, Beisson F, Andersson MX, Arondel V, Bates PD, Baud SEB, Bird D, DeBono A, Durrett TP, et al. Acyl-lipid metabolism. Arabidopsis Book. 2013;11(8):e133. 10.1199/tab.0161 PMC3563272 23505340

[50] Breiman L (2001). Random forests. Mach Learn.

[51] Kuhn M (2008). Building predictive models in R using the caret package. J Stat Softw.

[52] Tanabata T, Shibaya T, Hori K, Ebana K, Yano M (2012). *SmartGrain*: high-throughput Phenotyping software for measuring seed shape through image analysis. Plant Physiol.

[53] Anderson MJ (2001). A new method for non-parametric multivariate analysis of variance: Non-parametric manova for ecology. Austral Ecol.

[54] Anderson MJ, Walsh DC (2013). PERMANOVA, ANOSIM, and the mantel test in the face of heterogeneous dispersions: what null hypothesis are you testing?. Ecol Monogr.

[55] Oksanen J, Kindt R, Legendre P, O’Hara B, Stevens MHH, Oksanen MJ, Suggests M (2007). The vegan package. Community Ecol Package.

[56] Elshire RJ, Glaubitz JC, Sun Q, Poland JA, Kawamoto K, Buckler ES, Mitchell SE (2011). A robust, simple genotyping-by-sequencing (GBS) approach for high diversity species. PLoS One.

[57] Torkamaneh D, Laroche J, Bastien M, Abed A, Belzile F (2017). Fast-GBS: a new pipeline for the efficient and highly accurate calling of SNPs from genotyping-by-sequencing data. BMC Bioinformatics.

[58] Martin M (2011). Cutadapt removes adapter sequences from high-throughput sequencing reads. EMBnet.journal.

[59] Kagale S, Koh C, Nixon J, Bollina V, Clarke WE, Tuteja R, Spillane C, Robinson SJ, Links MG, Clarke C (2014). The emerging biofuel crop *Camelina sativa* retains a highly undifferentiated hexaploid genome structure. Nat Commun.

[60] Purcell S, Neale B, Todd-Brown K, Thomas L, Ferreira MA, Bender D, Maller J, Sklar P, De Bakker PI, Daly MJ, Sham PC. PLINK: a tool set for whole-genome association and population-based linkage analyses. Am J Hum Genet. 2007;81(3):559-75. 10.1086/519795 PMC1950838 17701901

[61] Alexander DH, Novembre J, Lang K (2009). Fast model-based estimation of ancestry in unrelated individuals. Genome Res.

[62] Behr AA, Liu KZ, Liu-Fang G, Nakka P, Ramachandran S (2016). Pong: fast analysis and visualization of latent clusters in population genetic data. Bioinformatics.

[63] Bates D, Sarkar D, Bates MD, Matrix L (2007). The lme4 package. R Package Version.

[64] Leroux SJ (2019). On the prevalence of uninformative parameters in statistical models applying model selection in applied ecology. PLoS One.

[65] Arnold TW (2010). Uninformative parameters and model selection using Akaike's information criterion. J Wildl Manag.

[66] Koller M. robustlmm: an R package for robust estimation of linear mixed-effects models. J Stat Software. 2016;75(6):1-24.

[67] Kuznetsova A, Brockhoff PB, Christensen RHB. lmerTest package: tests in linear mixed effects models. J Stat Software. 2017;82(13).

