# Supplementary figures and images for: Interactions between genetics and environment shape Camelina seed oil composition
Source: BMC Plant Biol. 2020 Sep 14;20:423. doi: 10.1186/s12870-020-02641-8 (PMC7490867; doi:10.1186/s12870-020-02641-8)

A

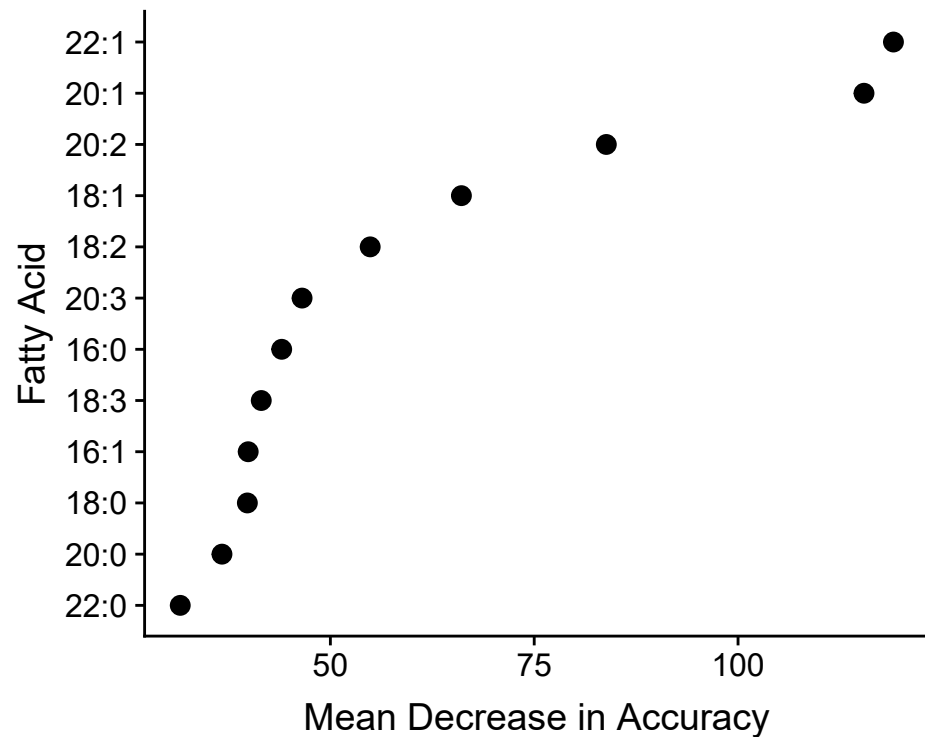

B

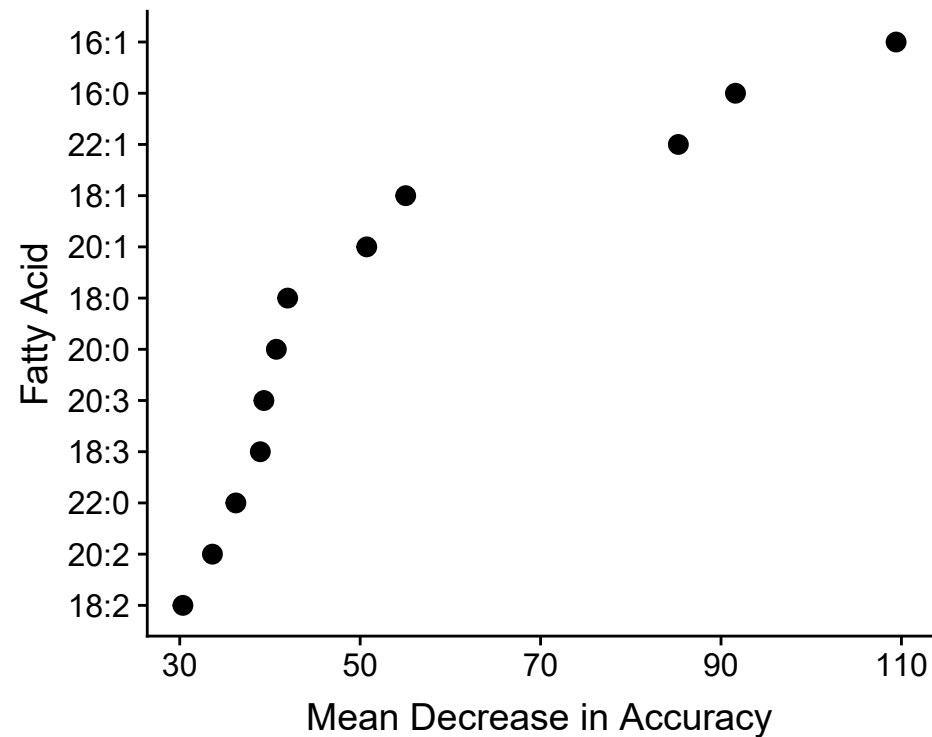

Supplement: Supplementary file 1 — Additional file 1: Figure S1. Random forest model importance plots for individual FAs comprising total seed oils in A) all Camelina species examined and B) populations of C. microcarpa. Mean decrease in accuracy is a measure of model accuracy decrease when individual factors are removed, with larger values indicative of more important factors. [file 12870_2020_2641_MOESM1_ESM.pdf]

Dimension 2

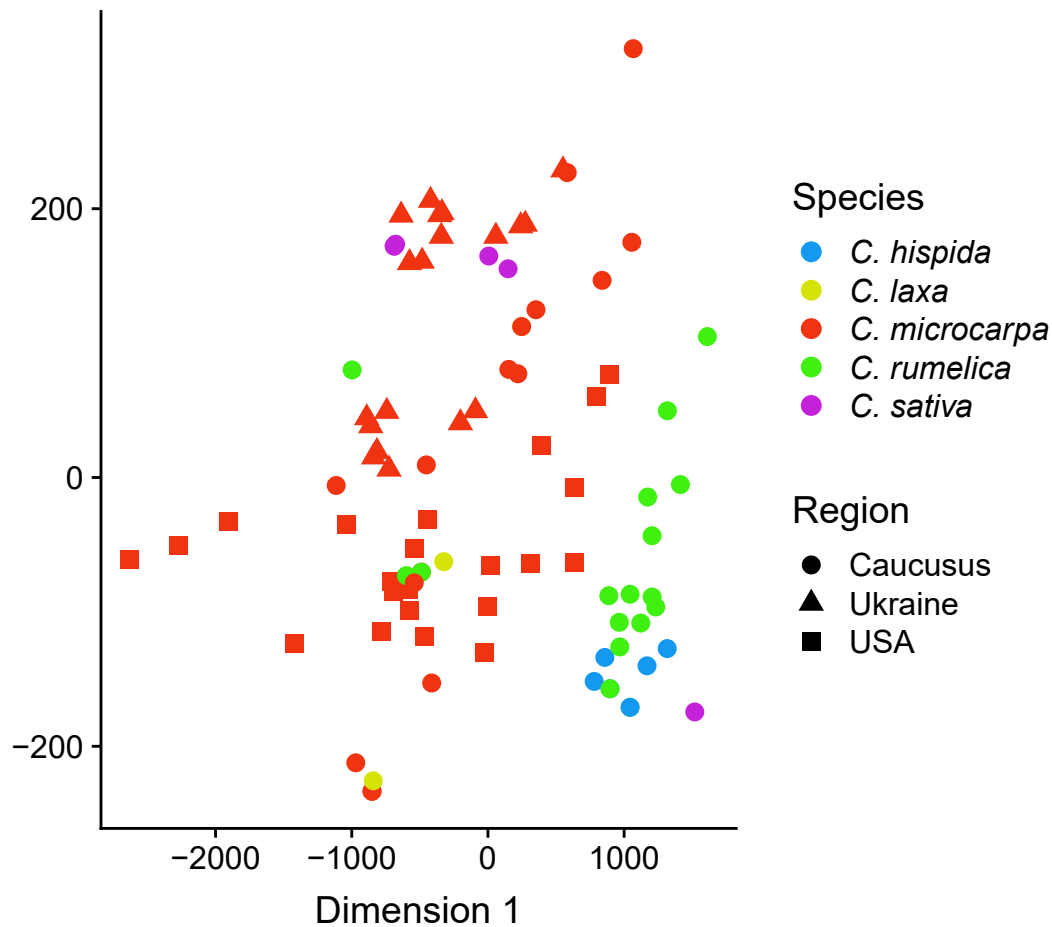

Dimension 1

Supplement: Supplementary file 3 — Additional file 3: Figure S3. NDMS plot of environments for different species of Camelina. Geographical locations of C. microcarpa individuals indicated with shapes as follows, circle = Caucasus, triangle = Ukraine, square = USA. Euclidean distance was used to construct the plot, stress = 0.01399579. [file 12870_2020_2641_MOESM3_ESM.pdf]

K = 1

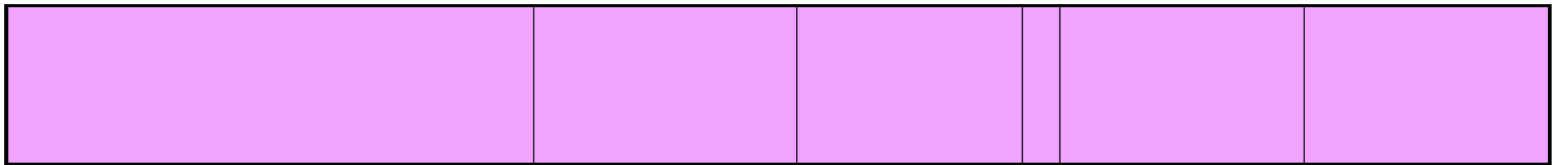

K = 2

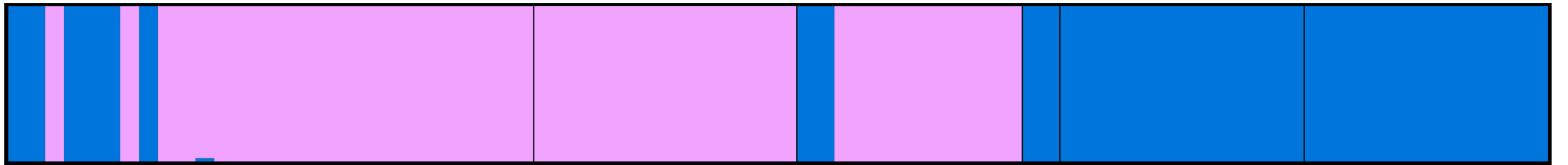

K = 3

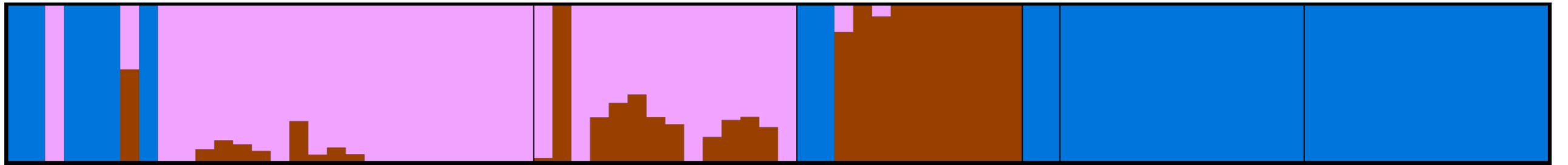

K = 4

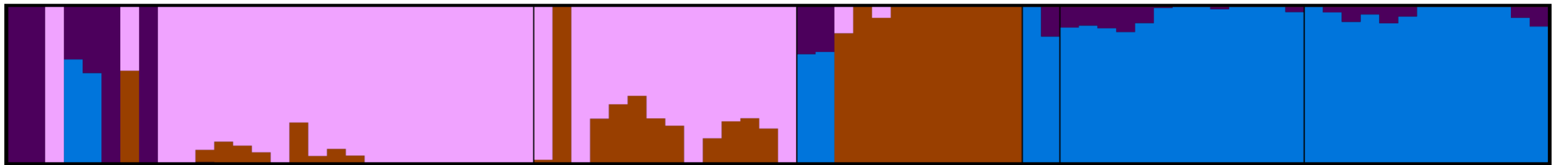

K = 5

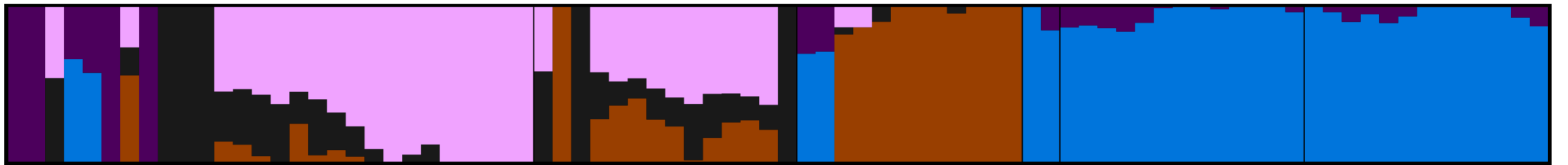

K = 6

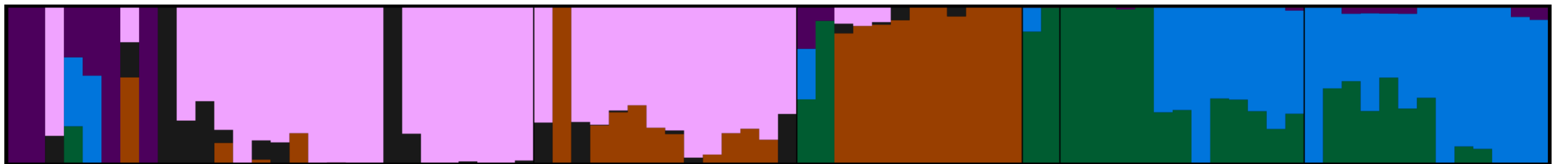

K = 7

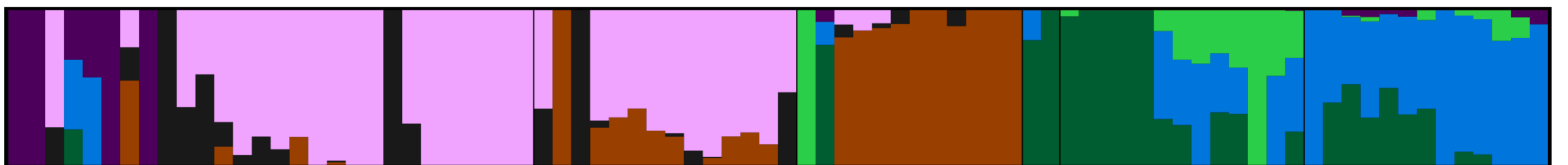

K = 8

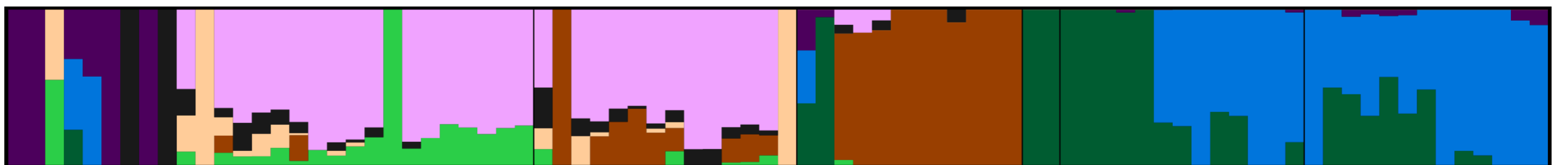

K = 9

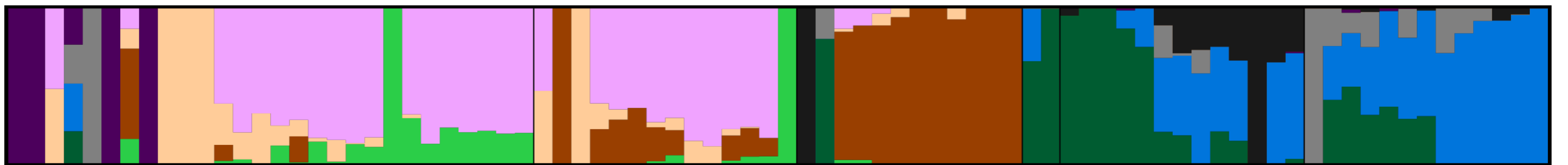

K = 10

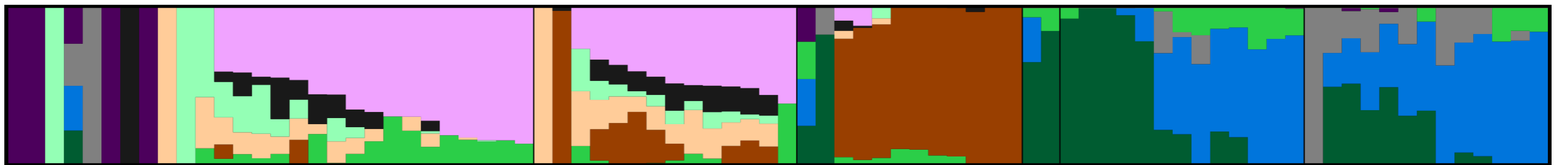

United States

N. Ukraine

S. Ukraine

Turkey

Armenia

Georgia

Supplement: Supplementary file 4 — Additional file 4: Figure S4. ADMIXTURE results displayed with pong at a range of K = 1–10, with 10 iterations per K. Individuals are divided into subgroups based on country of origin. [file 12870_2020_2641_MOESM4_ESM.pdf]

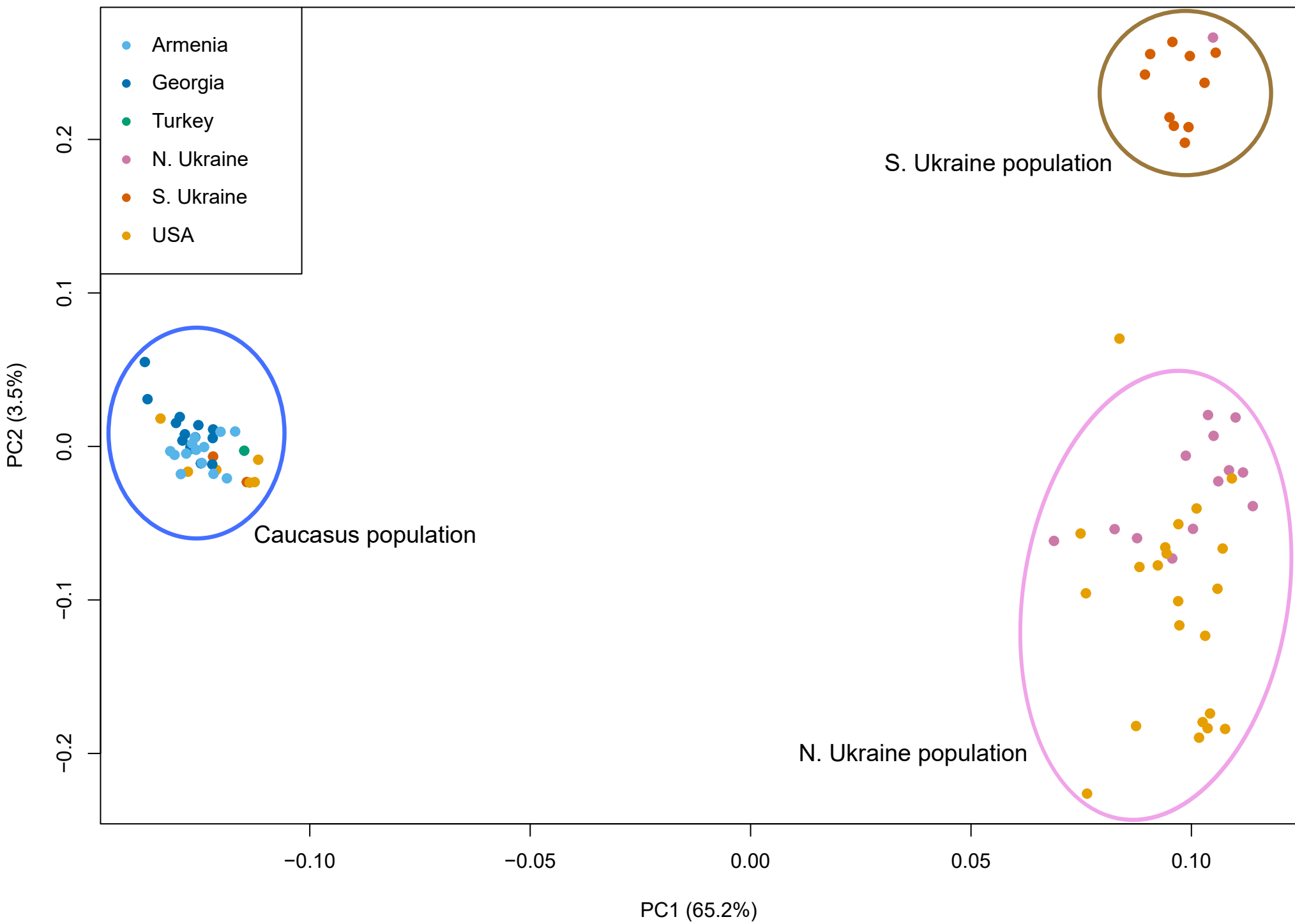

Supplement: Supplementary file 5 — Additional file 5: Fig\ure S5. PCA generated from the final SNP dataset of C. microcarpa accessions used in ADMIXTURE analyses. Colored dots represent individual’s country of origin, colored ellipses represent population identities as determined from ADMIXTURE. [file 12870_2020_2641_MOESM5_ESM.pdf]

standardized PC2 (25.6% explained var.)

standardized PC1 (47.4% explained var.)

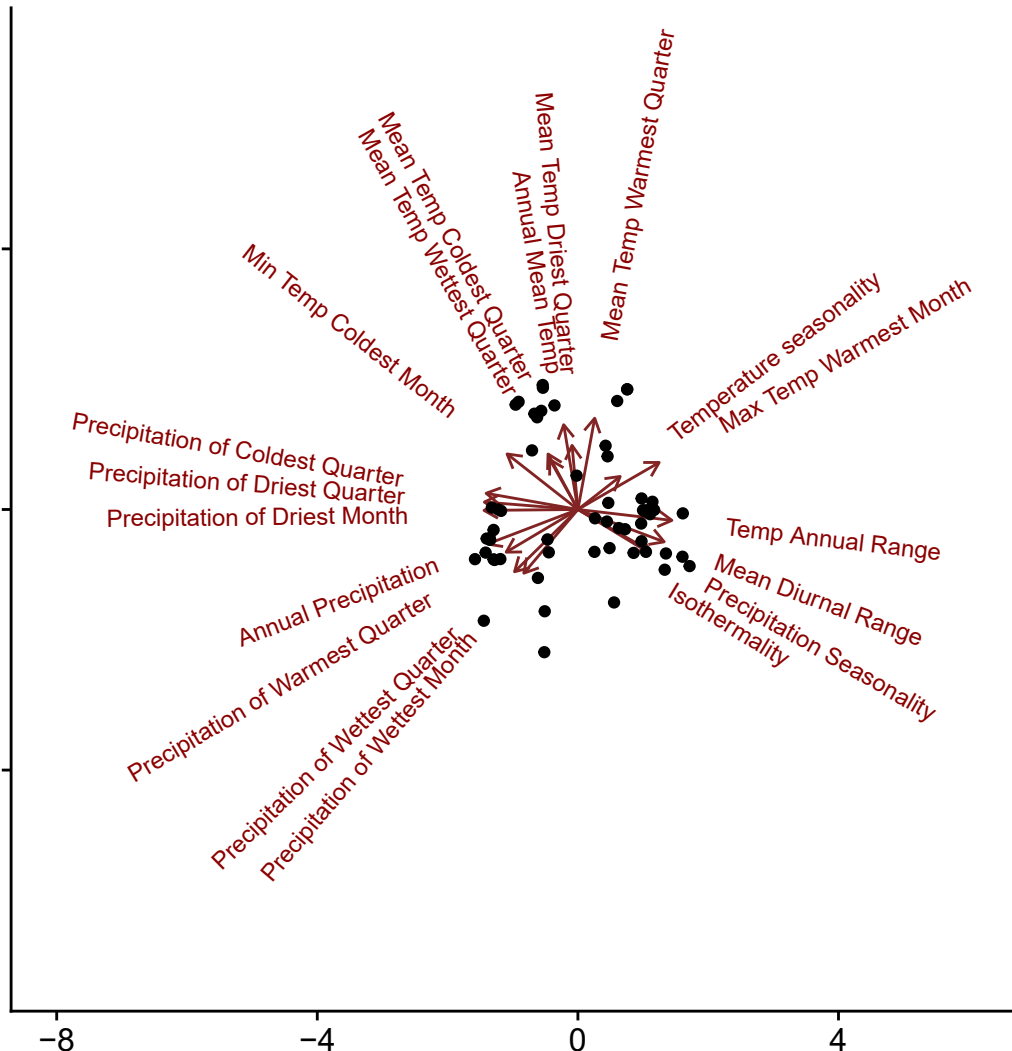

Supplement: Supplementary file 6 — Additional file 6: Figure S6. Box and whisker plot of fatty acid abundances detected in C. microcarpa genetic populations determined via gas chromatography. [file 12870_2020_2641_MOESM6_ESM.pdf]

*C. microcarpa*

Caucasus  
N. Ukraine  
S. Ukraine

Abundance (%)

40

30

20

10

0

Fatty Acid

16:0

16:1

18:0

18:1

18:2

18:3

20:0

20:1

20:2

20:3

22:0

22:1

S/U

Total

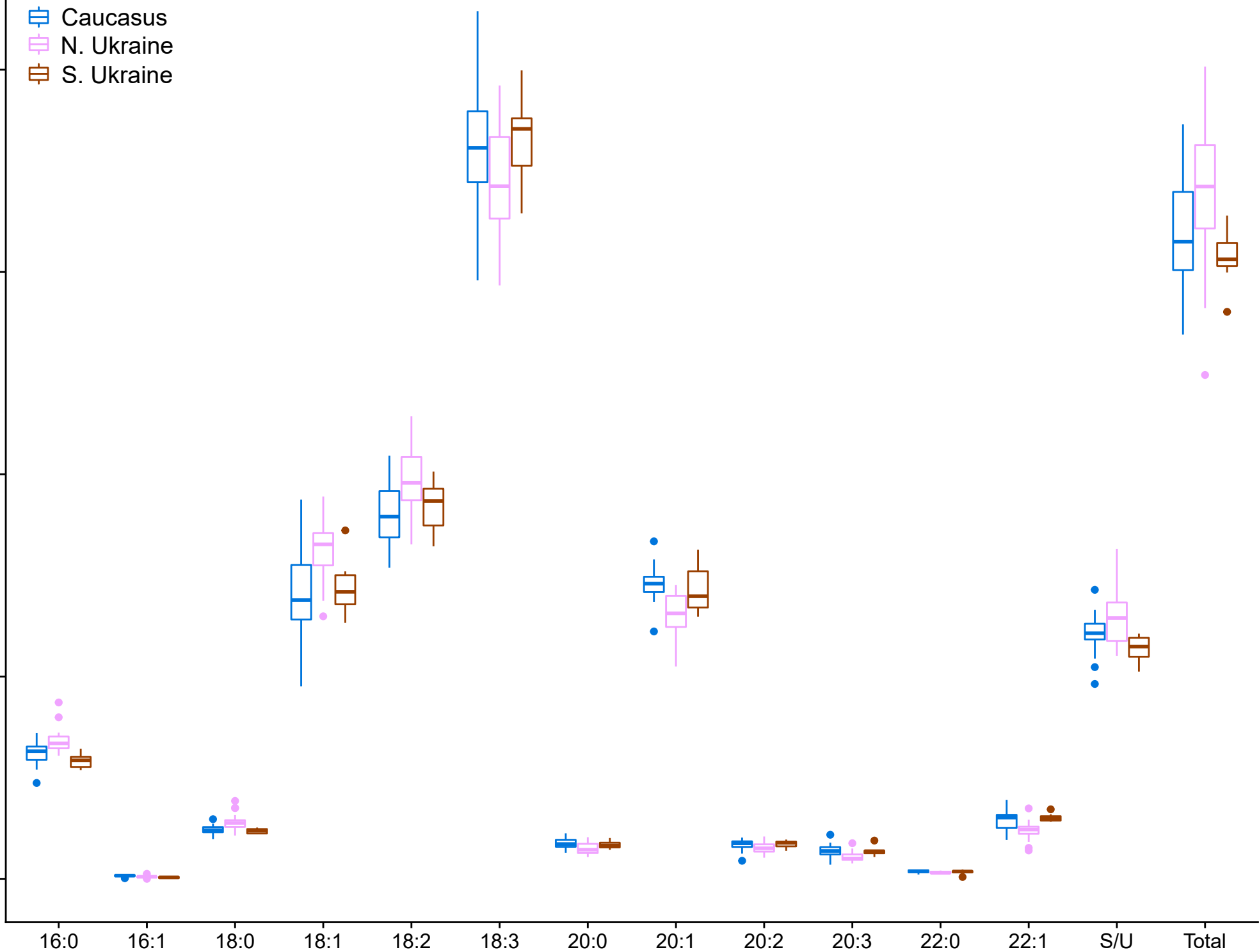

Supplement: Supplementary file 7 — Additional file 7: Figure S7. PCA generated from 19 BioClim variables determined from GPS coordinates for all accessions of Camelina in which seed oil composition was also analyzed. Red arrows are drawn according to weight and direction of effect for each climatic variable. [file 12870_2020_2641_MOESM7_ESM.pdf]

## Controlled Environment Growth Trial of *C. sativa* Accessions

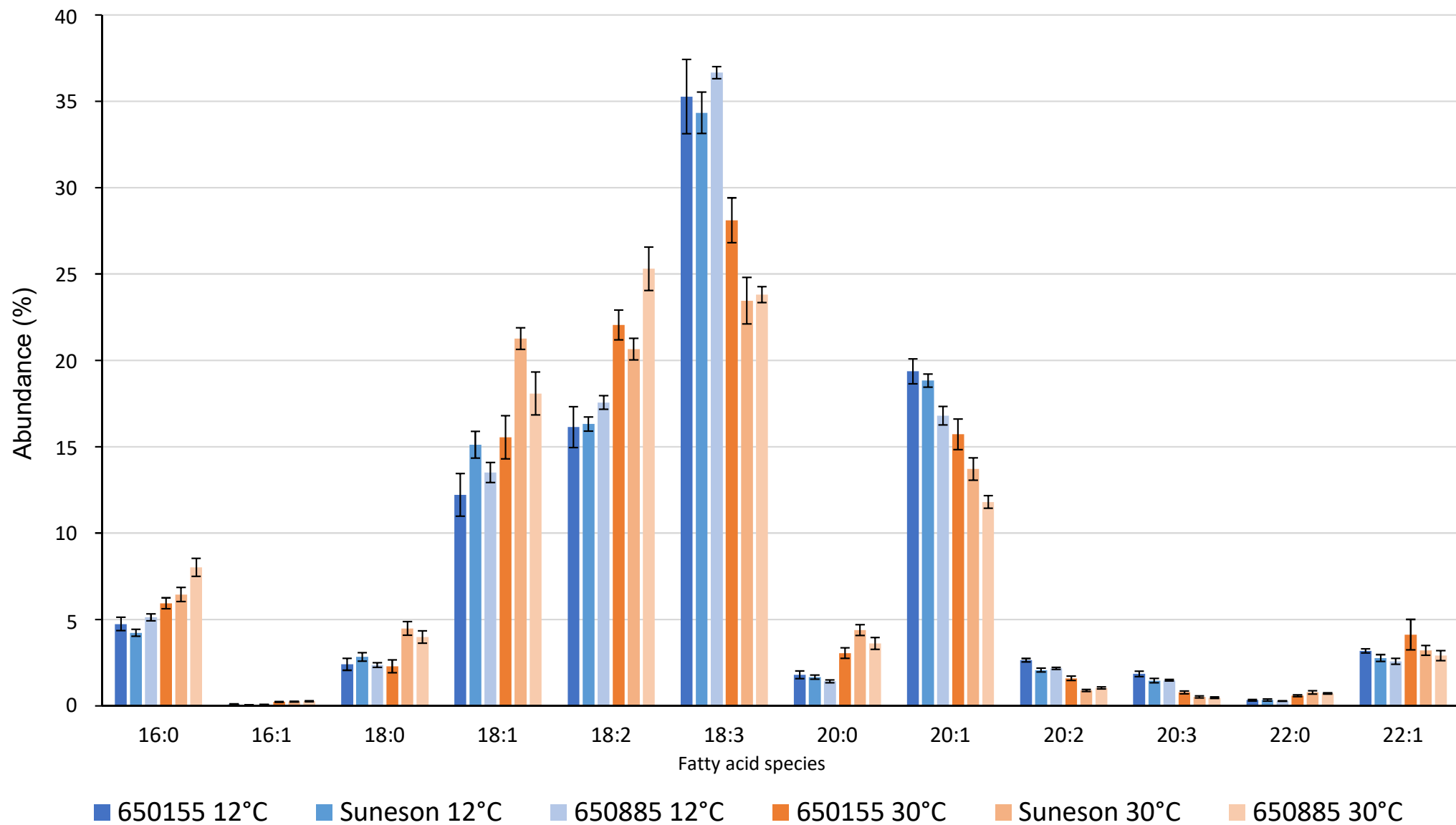

Supplement: Supplementary file 8 — Additional file 8: Figure S8. Fatty acid composition of three C. sativa accessions in 12 °C (blue) and 30 °C (orange) growth conditions as inferred by GC-MS. Error bars ±1 SD (n = 9). [file 12870_2020_2641_MOESM8_ESM.pdf]
