# Supplementary material for: Interactions between genetics and environment shape Camelina seed oil composition
Source: BMC Plant Biol. 2020 Sep 14;20:423. doi: 10.1186/s12870-020-02641-8 (PMC7490867; doi:10.1186/s12870-020-02641-8)
Supplement: Supplementary file 2 — Additional file 2: Figure S2. Confusion matrix from random forest model predictions of species identity based on FA composition for all Camelina species. Colored boxes represent the number of new observations categorized by the random forest model, such that correct determinations are represented along the diagonal, all other cells indicate incorrect determinations. Boxes are shaded such that boxes which are darker blue represent a higher proportion of the total number of samples for each species. [file 12870_2020_2641_MOESM2_ESM.pdf]

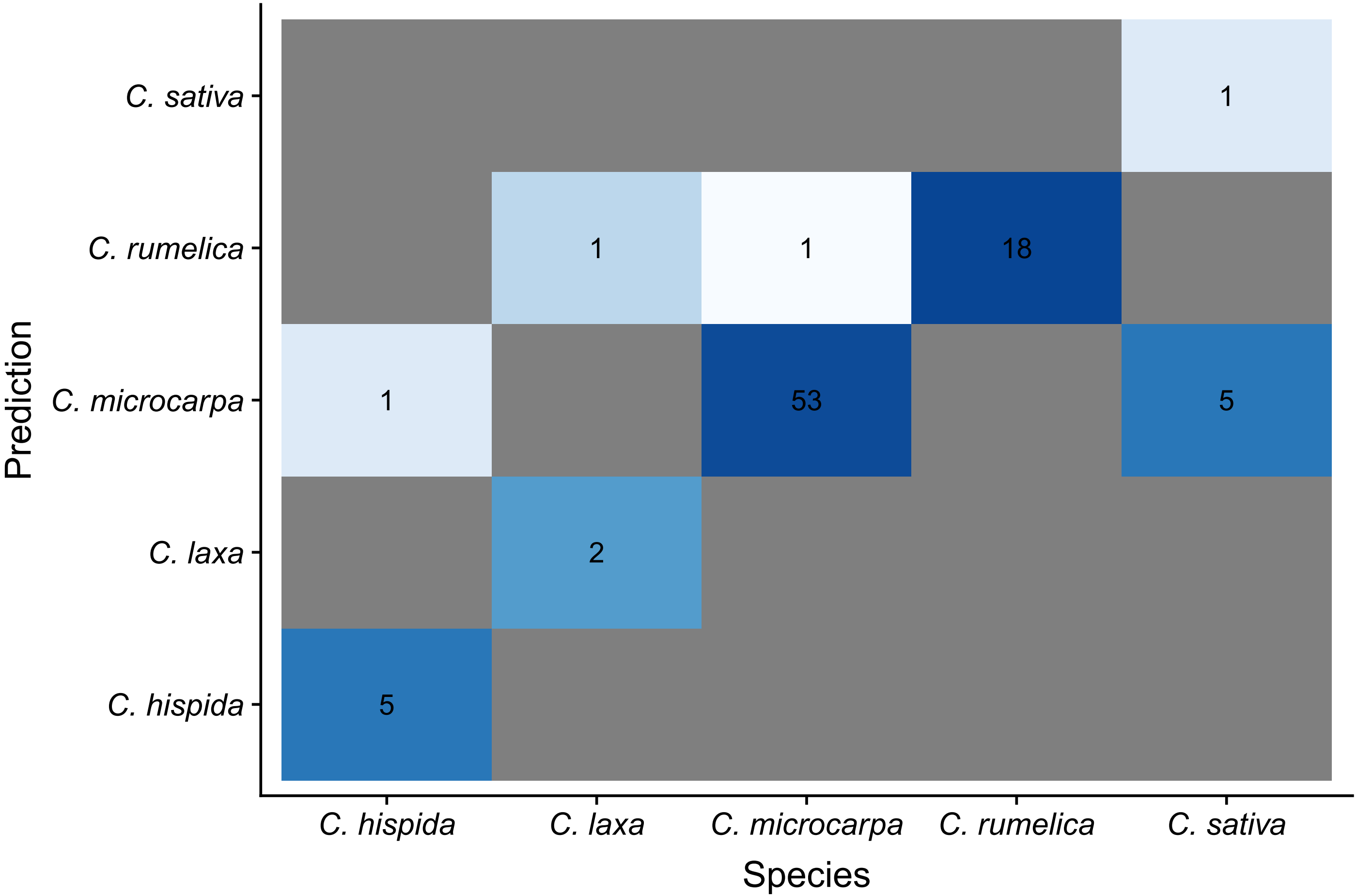
Prediction
C. sativa
C. rumelica
C. microcarpa
C. laxa
C. hispida
1
1
1
18
1
53
5
2
5
C. hispida
C. laxa
C. microcarpa
C. rumelica
C. sativa
Species
